# Mean Arterial Pressure Augmentation for Acute Traumatic Spinal Cord Injury: A Systematic Review and Meta-Analysis of Neurological Recovery and Mortality

**DOI:** 10.1177/21925682261458879

**Published:** 2026-06-05

**Authors:** Kush M. Kale, Shaan Patel, Shiva A. Nischal, Lorenzo Ceccon, Joshua Heller, Jack Jallo, James S. Harrop, Srinivas K. Prasad

**Affiliations:** 1 Department of Physiology, Anatomy & Genetics, Medical Sciences Division, 6396 University of Oxford, Oxford, UK; 2 Department of Neurological Surgery, 23217 Thomas Jefferson University Hospital, Philadelphia, PA, USA

**Keywords:** hemodynamic management, mean arterial pressure, neurocritical care, neurological recovery, spinal cord blood flow, spinal cord injury, spinal cord perfusion pressure

## Abstract

**Study Design:**

Systematic review.

**Objectives:**

Maintenance of mean arterial pressure (MAP) at 85-90 mmHg after traumatic spinal cord injury (SCI) is widely recommended, but supporting evidence is limited. We performed a systematic review and meta-analysis to evaluate whether MAP augmentation improves outcomes after SCI.

**Methods:**

PubMed, Embase, and CENTRAL were searched through January 2026. Random-effects meta-analyses were performed. Prespecified cohort-level analyses compared standard (≥65 mmHg) with augmented MAP targets (≥85 mmHg). Weighted linear meta-regression explored dose-response relationships between achieved MAP and primary outcomes.

**Results:**

Thirty-three studies comprising 3535 patients were included. Neurological improvement occurred in 31.4% and 37.6% of patients subjected to standard and augmented MAP, respectively (*P* = 0.25; I^2^ = 84.6). Mortality occurred in 38.8% and 10.3% of patients, respectively (*P* = 0.08; I^2^ = 83.9), though this was observed with markedly asymmetric denominators (103 vs 1162) and substantial inter-study heterogeneity, and is unlikely to reflect a true treatment effect. Respiratory complications were significantly lower in patients subjected to standard MAP (*P* = 0.04; I^2^ = 82.2%). Meta-regression did not identify a linear association between achieved MAP and either primary outcome. Certainty of evidence was low.

**Conclusions:**

Across heterogeneous, predominantly observational cohorts, pooled analyses showed no statistically significant differences in neurological improvement or mortality between standard and augmented MAP targets after acute traumatic SCI. A higher incidence of respiratory complications in patients subjected to augmented MAP represented the only statistically significant signal. These findings support avoidance of hypotension but do not establish clear benefit of universal MAP escalation.

## Background

Acute traumatic spinal cord injury (SCI) is a catastrophic neurological condition associated with profound and often permanent disability, substantial reductions in quality of life, and a major societal and economic burden.^1^ Contemporary Global Burden of Disease estimates suggest that over 20 million individuals worldwide are living with SCI, accounting for over 6 million years lived with disability, with incidence projected to rise further as populations age and trauma exposure increases.^2^ Despite advances in experimental neurobiology and surgical care, no disease-modifying therapy has been shown to reliably improve neurological recovery, placing heightened importance on optimization of acute neurocritical care strategies.

Hemodynamic management, specifically augmentation of mean arterial pressure (MAP), has emerged as a cornerstone of early SCI management, predicated on the rationale that improving spinal cord perfusion pressure (SCPP) may attenuate secondary ischemic injury. This rationale is grounded in experimental evidence that traumatic injury impairs spinal cord autoregulation, rendering local perfusion increasingly dependent on systemic arterial pressure and underpinning the principle that hypotension must be strictly avoided in the acute phase.^3^ Current clinical practice guidelines recommend maintaining MAP between 85 and 90 mmHg for 5-7 days following injury, typically using intravenous fluids and vasopressor therapy.^4^ These recommendations, however, are largely derived from small observational cohorts and expert consensus rather than high-quality comparative evidence, and the magnitude of their effect on clinically meaningful outcomes remains uncertain.^5,6^

Prior reviews^7-9^ have examined the role of blood pressure management in acute SCI, but most have been limited by narrative synthesis, inclusion of heterogeneous outcome definitions, or lack of quantitative pooling. A recent meta-analysis suggests potential associations between MAP targets and adverse outcomes, yet substantial methodological limitations (including inconsistent risk of bias assessment and limited exploration of dose-response relationships) restrict the interpretability of these findings.^9^ In addition a recent randomized clinical trial evaluating MAP targets in acute traumatic SCI has been published, providing new comparative evidence not incorporated in prior syntheses.^10^

Given the pervasive clinical adoption of MAP augmentation despite persistent uncertainty, a comprehensive and methodologically rigorous synthesis of the available evidence is warranted. We therefore conducted a systematic review, meta-analysis, and dose-response meta-regression to evaluate the association between MAP targets and measured MAP values with neurological recovery and mortality following acute SCI. By integrating data from randomized and observational studies and critically appraising study quality, this review aims to clarify whether MAP augmentation confers measurable clinical benefit or primarily exposes patients to additional hemodynamic risk without altering neurological trajectory.

## Methods

This systematic review and meta-analysis was conducted in accordance with the Preferred Reporting Items for Systematic Reviews and Meta-Analyses (PRISMA) guidelines^11^ and AMSTAR-2 (A MeaSurement Tool to Assess systematic Reviews 2) methodological standards. AMSTAR-2 criteria^12^ were used to assess the quality of systematic reviews published prior to this review. The study protocol was registered a priori on the Prospective Register of Systematic Reviews (PROSPERO) (ID: CRD420261278655).

### Search Strategy and Selection Criteria

We systematically searched PubMed, Embase, and CENTRAL from inception to 5^th^ January 2026 (Supplementary Table 1). No language restrictions were imposed. The reference lists of the included studies and relevant reviews were examined to identify additional studies.

Two reviewers independently screened the records using Rayyan,^13^ and the full texts were subsequently assessed for inclusion. Disagreements were resolved by consensus discussion with a third reviewer. Inter-rater reliability was calculated using Cohen’s kappa score (κ = 0.92), indicating an almost perfect agreement between the reviewers.

We included randomized and non-randomized (observational) studies reporting at least 1 predefined outcome of interest and including acute SCI patients subjected to a MAP threshold or undergoing MAP measurement. Observational studies without a comparator group were eligible and were analyzed using single-arm meta-analytic methods, reflecting the structure of the available evidence. Exclusion criteria were case reports, case series, reviews, conference abstracts, technical notes, and animal studies.

### Outcomes of Interest

Primary outcomes were American Spinal Injury Association (ASIA) impairment scale (AIS) improvement (defined as the proportion of patients achieving at least 1 grade of AIS improvement, or the mean number of AIS grades by which patients improved, at the first follow-up) and mortality.

Secondary outcomes were as follows: (i) overall complications (defined as the proportion of patients experiencing at least 1 post-injury complication); (ii) system-specific complications (respiratory, cardiovascular, and renal); (iii) length of stay (LOS), including length of hospital stay and intensive care unit (ICU) stay.

### Data Extraction

Data from studies fulfilling our inclusion criteria were independently extracted by 2 reviewers. Study characteristics (author, date, country, design), sample size, patient demographics, follow-up duration, MAP threshold, measured MAP, and clinical outcomes of interest were extracted from the studies.

### Statistical Analysis

Study-level meta-analyses were conducted for outcomes with sufficient (reported by at least 2 studies) and compatible data. Continuous and dichotomous outcomes were pooled as mean differences (MDs) and risk ratios (RRs), respectively. Given very few of the included studies reported both a standard- and an augmented-MAP arm, pooled within study RRs could not be meaningfully calculated for primary outcomes – comparisons between cohorts therefore relied on the cohort-level analyses described above. A random-effects model using the restricted maximum likelihood (REML) estimator was applied to all outcomes. For single-arm binary outcomes, we extracted the number of participants experiencing ≥1 event and the total at risk, and synthesized pooled random-effects proportions with 95% confidence intervals (CIs). Proportions were meta-analyzed on the logit scale using inverse-variance weighting (R package *meta*; *metaprop* with logit transformation and REML). For comparative dichotomous outcomes, we calculated study-specific effect estimates and pooled risk ratios (RRs). For continuous outcomes, we pooled the mean differences (MDs). Prespecified cohort-level comparisons were performed by mean arterial pressure (MAP) target, categorizing studies as standard MAP (≥65 to <85 mmHg) or augmented MAP (≥85 mmHg; typically 85-90 mmHg) according to each study protocol; where sufficient data were available, we generated separate random-effects pooled estimates for each pooled cohort and displayed these in forest plots. Prespecified sensitivity analyses were performed by injury level (cervical-only vs mixed-level), and by injury severity (complete injury (AIS A)-only vs mixed severity). All analyses were performed using the *meta* package^14^ in R version 4.3.2 (R Foundation for Statistical Computing).^15^

Heterogeneity was assessed using Cochran’s Q (*P* < 0.10 considered statistically significant) and quantified using I^2^ (≥25% considered non-trivial). We additionally report τ from the REML model. Small-study effects were evaluated using funnel plots when ≥10 studies contributed to an outcome.

To explore a potential dose–response relationship between mean arterial pressure (MAP) and neurological (AIS) improvement and mortality, we performed a weighted study-level linear meta-regression. For each study, we extracted the mean measured MAP during the acute management period, outcome data (events and total sample size for binary outcomes; mean and standard deviation for continuous outcomes), and prespecified study-level covariates (mean age, proportion of men, proportion with AIS grade A injury, and proportion with cervical injury). For binary outcomes, we modelled the logit-transformed event proportion as the dependent variable with mean MAP as the primary predictor, weighted by the total study sample size. For continuous outcomes, we modelled study-specific mean differences as the dependent variable, again weighting by the total study sample size. The exploratory adjusted models additionally included age, sex, AIS A, and cervical proportions as covariates. Adjusted associations were visualized using bubble plots overlaid with the covariate-adjusted regression line (predicted at mean covariate values).

### Risk of Bias and Certainty of Evidence

Two authors independently evaluated the certainty of evidence for each outcome using the BMJ Grading of Recommendations Assessment, Development and Evaluation (GRADE) framework^16^ and the risk of bias using the Cochrane’s Risk of Bias 2 (RoB 2) tool^17^ for randomized studies and Risk of Bias in Non-Randomised Studies of - Interventions tool (ROBINS-I)^18^ for non-randomized studies. Disagreements were resolved through consensus discussions with a third reviewer. The overall risk of bias was determined by aggregating domain-level judgements.

## Results

**Figure 1.**
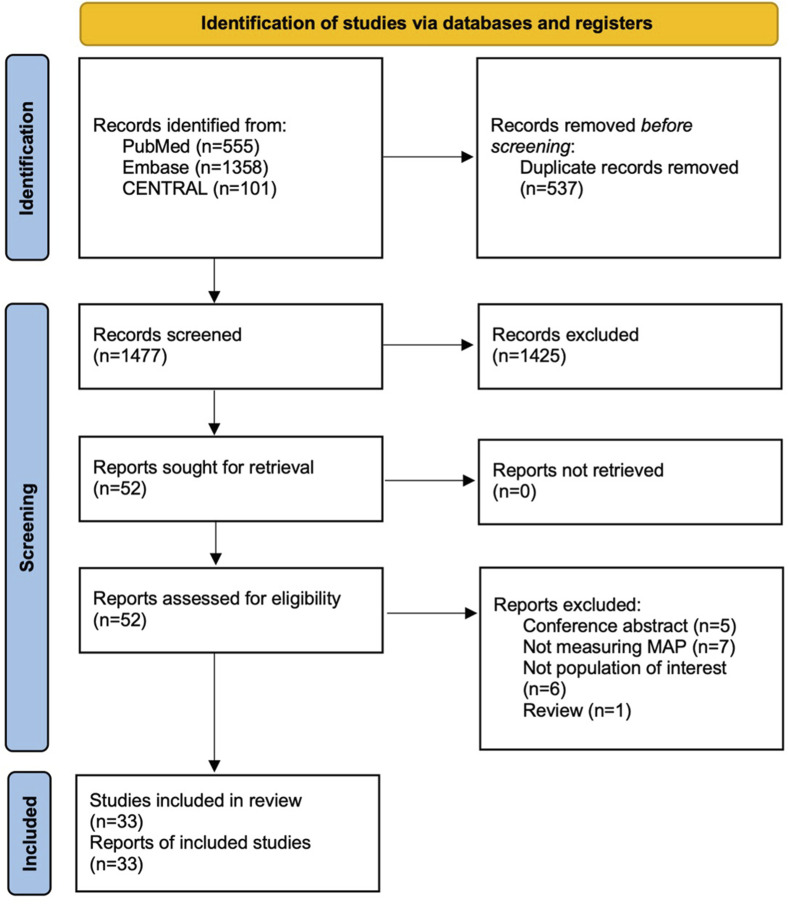
PRISMA flow diagram of study selection. Preferred reporting items for systematic reviews and meta-analyses (PRISMA) flow diagram outlining the study selection process. The number of records identified, screened, assessed for eligibility, and included in the final analysis are detailed at each stage

Our initial search identified 1477 records, of which 33 met the inclusion criteria (Figure 1).

These comprised 28 retrospective observational studies, 4 prospective observational studies, and 1 randomized controlled trial (RCT), collectively comprising 3535 patients (Table 1). Study sample sizes ranged from 17 to 801 participants. Reported mean ages ranged from 32.8 to 61.5 years. The proportion of male patients ranged from 63.3% to 89.8%. The proportion of ASIA grade A injuries ranged from 2.5% to 100.0%. Duration of follow-up ranged from 5.0 to 593.7 days. Among studies reporting achieved MAP, mean values were 81.7 mmHg in standard-target cohorts and 90.0 mmHg in augmented (Supplementary Table 2). Vasopressor use was heterogeneous, most commonly dopamine (17 studies), norepinephrine (14 studies), and phenylephrine (12 studies), though most studies used multiple. Timing of neurological outcome assessment (ASIA) ranged from 5.0 to 365.0 days post-injury (Table 1).

**Table 1.** Characteristics of Included Studies

Author (Year)	Country	Study design	Sample size (n)	Age, in years (mean ± SD or mean (range))	Sex (% male)	Injury level (n)	AIS on admission (n)	ASIA motor score on admission	ASIA motor score at discharge	Neurological assessment timing post-injury, in days (mean ± SD or range)	Injury mechanism (n)	Received vasopressor (n/N)	Choice of vasopressor	MAP target (mmHg)	Mean MAP (mmHg)	MAP target duration (days)	Duration of follow-up (days) (mean ± SD or mean (range))
Agarwal (2022)	USA	RCS	74	47.3 ± 26.7	73.0%	NR	A: 37; B: 8; C: 14; D: 13; E: 2	NR	NR	At discharge (duration NR)	Penetrating: 5; hemorrhagic: 5; central cord: 24	NR	Dopamine; phenylephrine	76-104	NR	NR	Until discharge
Alfin (2023)	Nigeria	RCS	21	36.0 ± 13.4	83.3%	Cervical: 139; cervicothoracic: 20; thoracic: 46; Thoracolumbar: 58; lumbar: 33	A: 185; B: 54; C: 21; D: 24; E: 12	NR	NR	42.0	Fall: 63; Gunshot: 20; MVA: 156; Occupational: 57	NR	NR	<80	89.0 ± 8.9	7	42.0
			86											80-90			
			157											90-100			
			32											>100			
Balasuberamaniam (2023)	Canada	RCS	82	50.6 ± 21.3	89.0%	Cervical: 48; thoracic: 34	A: 82	NR	NR	NR	NR	82/82	Norepinephrine; Epinephrine; Vasopressin, phenylephrine; dopamine	>80	NR	NR	Until discharge
Catapano (2017)	USA	RCS	62	46.4 ± 21.1	NR	Cervical: 40; thoracic: 17; lumbar: 4	A: 33; B or C: 17; D: 12	NR	NR	At discharge (28.0 ± 14.2)	Penetrating: 10	NR	Dopamine; phenylephrine	>85	94.6 ± 2.8	3	Until discharge
Cohn (2010)	USA	RCS	17	32.8 ± 22.0	82.4%	Cervical: 17	A: 17	19.8 ± 14.2	24.4±15.3	At discharge (22.0 ± 22.0)	MVA: 6; Fall: 3; diving: 2; sport: 3; other: 3	4/17	Dopamine; phenylephrine	>65	NR	7	Until discharge
Dakson (2017)	Canada	RCS	94	47.6 ± 20.7	78.7%	Cervical: 66; Thoracolumbar: 28	A: 31; B: 12; C: 16; D: 29; NR: 6	44.9 ± 28.2	NR	At discharge (26.7±19.5)	MVA: 26; ATV: 11; Fall: 45; other: 12	NR	Norepinephrine	>70	NR	5	252.0±152.8
Ehsanian (2020)	USA	RCS	25	42.4 ± 17.7	NR	Cervical: 19; thoracic: 6	A: 11; B: 4; C: 3; D: 7	NR	NR	At discharge (31.3 ± 18.5)	Sports: 8; MVA: 6; Fall: 10; other traumatic: 1	24/25	Phenylephrine; Ephedrine; dopamine; norepinephrine; Vasopressin	>80	78.3 ± 6.5	NR	Until discharge
Haldrup (2020)	Denmark	RCS	129	50.0 (20.0-80.0)	81.4%	Cervical: 50; thoracic: 46; lumbar: 33	A: 58; B: 10; C: 24; D: 37	NR	NR	365.0	NR	NR	Metaoxedrin; norepinephrine	>80	75.8 ± 12.6	7	365.0
Hawryluk (2015)	USA	RCS	71	46.7 ± 20.3	74.6%	Cervical: 46; thoracic: 17; lumbar: 3	A: 29; B: 5; C: 13; D: 18; E: 3	NR	NR	At discharge (30.0 ± 35.5)	Penetrating: 10	71/71	Phenylephrine; dopamine	>85	94.0 ± 1.2	5	30.0
Inoue (2014)	USA	RCS	131	41.3 ± 22.2	74.2%	Cervical: 101; thoracic: 30	A: 66; B to E: 62	NR	NR	NR	NR	131/131	Dopamine; phenylephrine	>85	NR	7	Until discharge
Jiang (2019)	Canada	RCS	801	46.0 (29.0-59.0)	76.0%	Cervical: 528; thoracic: 166; lumbosacral: 50	A: 231; B: 72; C: 90; D: 175; E: 46	NR	NR	180.0	Fall: 316; MVA: 298; other: 181	NR	NR	>85	NR	NR	365.0
Kepler (2015)	USA	RCS	92	54.4 ± 25.0	72.8%	Cervical: 57; cervicothoracic: 16; thoracic: 5; Thoracolumbar: 4; C + L: 2; C + T + L: 6; T + S; 1	A: 27; NR: 65	33.7 ± 26.9	NR	5.0	Fall: 51; MVA: 32; other: 5	NR	NR	>85	NR	5	5.0
Langsjo (2024)	Finland	RCS	19	52.7 ± 22.2	89.5%	Cervical: 19	A: 7; B: 4; C: 4; D: 4	NR	NR	55.2 ± 22.8	NR	NR	Norepinephrine	65-85	81.6 ± 3.9	7	110.0 (47.0-136.0
			32	56.6 ± 15.7	84.4%	Cervical: 32	A: 8; B: 5; C: 12; D: 7	NR	NR	84.2 ± 30.7	NR	NR		85-90	90.2 ± 2.2		69.0
LaRiccia (2023)	USA	RCS	96	52.4 ± 18.2	77.1%	NR	A: 28; B: 14; C: 18; D: 36	NR	NR	At discharge (13.5 ± 9.6)	Blunt	79/96	Norepinephrine	>85	NR	4	Until discharge
LaRiccia (2025)	USA	PCS	164	53.1 ± 20.8	72.0%	NR	A: 51; B: 22; C: 38; D: 41; E: 12	NR	NR	At discharge (13.7 ± 10.8)	Blunt	NR	NR	>85	97.1 ± 17.7	7	Until discharge
Levi (1993)	USA	RCS	50	39.7 (17.0-78.0)	88.0%	Cervical: 50	NR	NR	NR	NR	Fall: 11; MVA: 25; Gunshot: 8; other: 6	41/50	Dopamine; dobutamine	>90	94.4 ± 9.4	7	42.0
Martin (2015)	USA	RCS	105	49.5 ± 20.6	70.5%	Cervical: 60; cervicothoracic: 20; thoracic: 25	NR	44.3 ± 26.9	NR	At discharge (18.3 ± 13.1)	Fall: 58; MVA 26; motorcycle: 12; other: 5; diving: 2; Gunshot: 2	70/105	NR	>85	85.0 ± 6.4	NR	Until discharge
Mishra (2021)	USA	RCS	80	41.4 ± 12.7	86.3%	Cervical: 80	A: 41; B: 7; C: 11; D: 7; E: 9	NR	NR	NR	NR	54/80	Dopamine; norepinephrine	>85	NR	7	Until discharge
Mushlin (2020)	USA	RCS	193	43.0 ± 19.0	81.9%	Cervical: 193	A: 114; B: 79	15.0 ± 12.8	NR	180.0	NR	NR	NR	>85	89.0 ± 5.7	7	593.7±106.8
Park (2016)	South Korea	RCS	73	44.2 (14.0-83.0)	79.5%	Cervical: 73	A: 36; B: 6; C: 12; D: 19; E: 0	NR	NR	7.0	NR	NR	NR	>85	NR	7	90.0
Rask (2024)	Sweden	RCS	52	53.0 ± 18.0	73.1%	Cervical: 52	A + B: 35; C + D: 17	NR	NR	90.0	MVA: 24; sport: 4; Fall: 23; other: 1	NR	Dobutamine; norepinephrine	>85	81.0±10.3	7	90.0
Readyy (2015)	USA	RCS	34	61.5 ± 16.3	82.4%	Cervical: 34	A: 8; B: 5; C: 8; D: 12; E: 1	NR	NR	At discharge (18.6 ± 19.1)	NR	34/34	Dopamine; phenylephrine	>85	NR	4	Until discharge
Readyy (2016)	USA	RCS	36	39.4 ± 19.2	77.8%	Cervical: 14; thoracic: 19; lumbosacral: 3	A: 36	NR	NR	At discharge (37.1 ± 35.7)	Penetrating: 14; blunt: 22	36/36	Dopamine; phenylephrine	>85	NR	4	Until discharge
Rerikh (2020)	Russia	RCS	110	34.3 ± 12.3	82.7%	Cervical: 59; thoracic: 28; lumbar: 23	A: 64; B: 12; C: 15; D: 19	NR	NR	120.0-180.0	NR	NR	Norepinephrine; dopamine; dobutamine	>85-90	NR	7-10	180.0
Sajdeya (2025)	USA	RCT	46	53.6 ± 17.8	82.6%	Cervical: 42;	A: 24; B: 9; C: 13	NR	UEMS: 35.0 ± 14.2; LEMS: 18.5 ± 14.2	180.0	Blunt	28/46	Phenylephrine; norepinephrine; Vasopressin; dopamine	>85-90	86.1 ± 11.9	7	180.0
			46	54.0 ± 17.9	82.6%	Cervical: 33	A: 26; B: 5; C: 14	NR	UEMS: 33.0 ± 15.9; LEMS: 20.0 ± 20.0			24/46		>65-70	87.9 ± 14.0		
Santos (2014)	Brazil	RCS	49	34.7 ± 17.2	89.8%	Cervical: 26; thoracic: 15; lumbar: 8	NR	NR	NR	NR	Fall: 18; Gunshot: 15; MVA: 12; stab: 3; other: 1	9/49	Norepinephrine; atropine; dopamine	NR	83.9	NR	Until discharge
Sewell (2019)	Australia	RCS	79	43.5 (16.0-83.0)	63.3%	Cervical: 52; thoracic: 27	A: 27; B: 28; C: 11; D: 13	NR	NR	NR	NR	14/79	NR	>80	NR	1	30.0
Squair (2019)	USA	PCS	92	43.0 ± 16.0	78.3%	Cervical: 55; thoracic: 28; lumbar: 9	A: 57; B: 18; C: 17	33.8±19.6	Total: 49.6 ± 26.7; UEMS: 36.7 ± 15.3; LEMS: 13.5 ± 18.5	180.0	NR	NR	Norepinephrine; phenylephrine; dopamine	>80-85	85.0 ± 6.0	5	180.0
Visagan (2023)	UK	PCS	13	44.2 ± 18.9	76.9%	Cervical: 13	A: 4; B: 7; C: 2	NR	NR	240.0	Blunt	NR	Norepinephrine	>85	NR	NR	180.0
Vale (1997)	USA	PCS	64	NR	84.4%	Cervical: 35; thoracic: 29	A: 31; B: 11; C: 9; D: 13	NR	NR	365.0	MVA: 46; Fall: 13; sport: 1; motorcycle: 1; diving: 2; other: 1	30/64	Dopamine; norepinephrine	>85	NR	7	365.0
Weinberg (2021)	USA	RCS	136	52.0 ± 19.0	75.0%	Cervical: 102; thoracic: 29; lumbar: 5	A: 30; B: 20; C: 28; D: 58	NR	NR	At discharge (10.4 ± 1.7)	Blunt	106/136	Norepinephrine; phenylephrine; dopamine; Vasopressin	>85	NR	3	Until discharge
Wolf (1991)	USA	RCS	52	37.2	78.9%	Cervical: 52	NR	NR	NR	NR	MVA: 28; Fall: 12; diving: 7; Occupational: 3; assault: 2	NR	NR	>85	NR	5	365.0
Zhang (2024)	China	RCS	40	54.7 ± 11.1	67.5%	Cervical: 40	A: 1; B: 5; C: 17; D: 17	NR	NR	NR	NR	NR	NR	>70	NR	NR	365.0

Summary of demographic and injury-related characteristics for all comparative studies included in the meta-analysis. Columns report study author and publication year, country of study origin, study design, total sample size, age distribution, sex distribution, injury level, admission AIS grade, injury mechanism, vasopressor use, MAP target, mean MAP, and monitoring duration. Abbreviations: RCS = retrospective cohort study; PCS = prospective cohort study; RCT = randomized controlled trial; NR = not reported; AIS = ASIA impairment scale; MAP = mean arterial pressure; MVA = motor vehicle accident; C + L = combined cervical and lumbar spinal cord injury; C + T + L = combined cervical, thoracic and lumbar spinal cord injury; T + S = combined thoracic and sacral spinal cord injury.

Methodological appraisal using AMSTAR-2 rated the present review as high quality (Supplementary Table 3).

### Neurological Improvement

**Figure 2.**
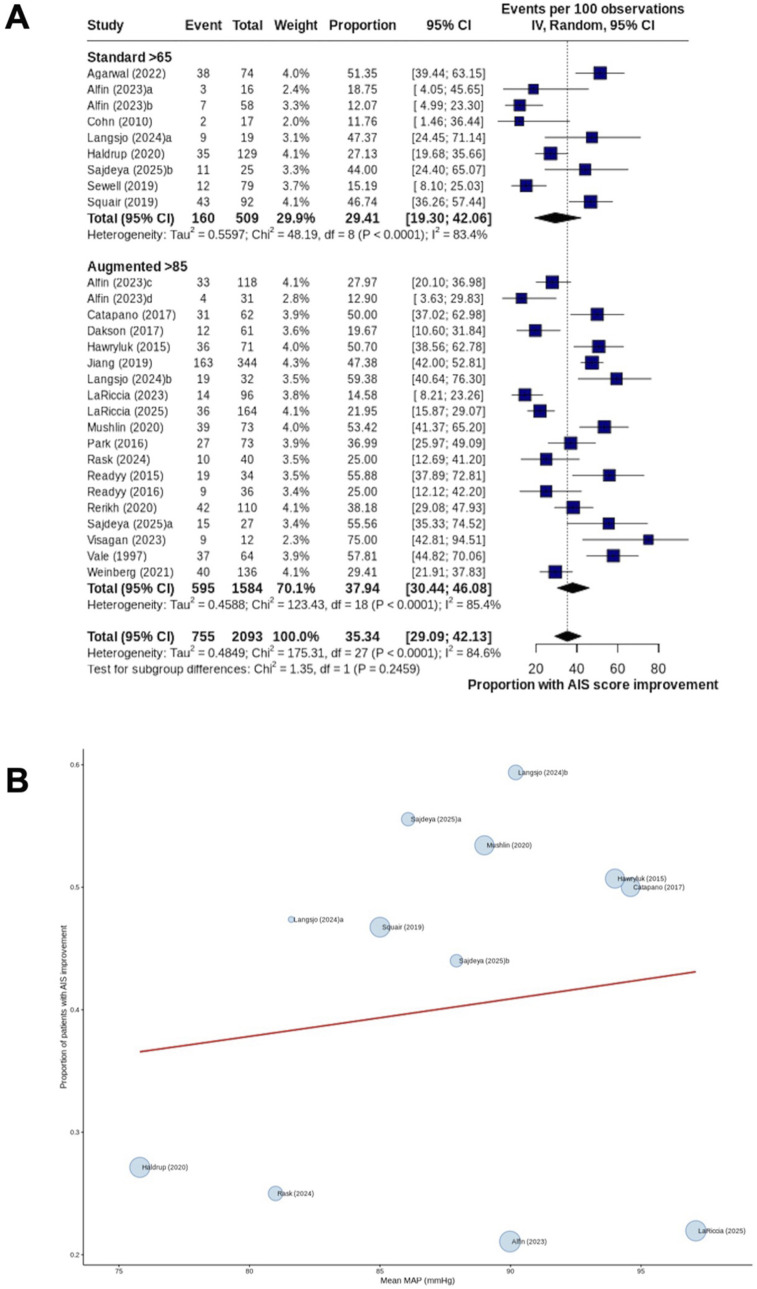
AIS improvement proportion (A) Forest plot comparing (random effects model) the proportion of patients experiencing AIS grade improvement between cohorts subjected to standard MAP threshold and those undergoing MAP augmentation. Individual study estimates are shown as squares proportional to study weight. Horizontal lines indicate 95% confidence interval. Diamond represents pooled estimate for overall effect. IV = inverse variance; (B) Dose-response meta-regression for AIS improvement proportion with MAP as the moderator

Twenty-three studies^6,10,19-39^ (69.7%) reported the proportion of patients who experienced AIS grade improvement. Improvement occurred in 31.4% of patients subjected to the standard MAP threshold (≥65 to <85 mmHg) (160/509 patients) and 37.6% of patients subjected to MAP augmentation (≥85 mmHg) (595/1584 patients). There was no significant difference between cohorts (*P* = 0.25; I^2^ = 84.6) (Figure 2A). Sensitivity analyses by treatment era (pre-vs post-2010) and duration of follow-up (restricted to studies reporting outcomes at ≥ 3 months post-injury) did not suggest an effect of these moderators (Supplementary Figures 1–2). Exploratory cohort-level comparisons stratified by injury level and severity demonstrated no significant differences (Supplementary Figure 3). Dose-response meta-regression across ten studies^10,20,21,24,25,27,28,30,32,37^ (30.3%) suggested that achieved MAP was not significantly associated with the proportion of patients experiencing AIS grade improvement (β = -80.007; 95% CI -0.167−0.153; *P* = 0.93) (Figure 2B).

**Figure 3.**
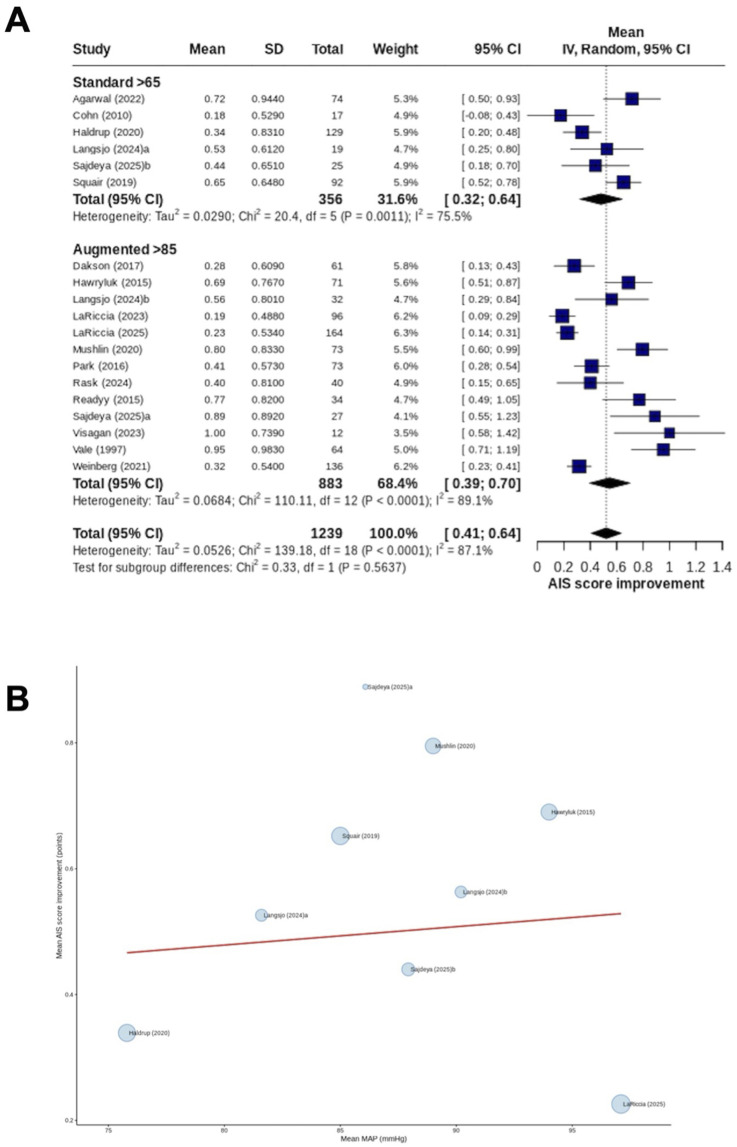
AIS mean improvement (A) Forest plot comparing (random effects model) the mean AIS grade improvement between patients subjected to standard MAP threshold and those undergoing MAP augmentation. Individual study estimates are shown as squares proportional to study weight. Horizontal lines indicate 95% confidence interval. Diamond represents pooled estimate for overall effect. IV = inverse variance; (B) Dose-response meta-regression for AIS mean improvement with MAP as the moderator

Mean AIS improvement was reported by seventeen studies^6,10,19,22-25,27-33,37-39^ (51.5%). The mean improvement was 0.48 grades in patients subjected to the standard MAP threshold (356 patients) and 0.55 grades in patients subjected to MAP augmentation (883 patients), with no significant cohort-level comparisons (*P* = 0.56; I^2^ = 87.1) (Figure 3A). Dose-response meta-regression across 7 studies^10,24,25,27,28,30,37^ (21.2%) suggested that MAP was not significantly associated with mean AIS grade improvement (β = 0.003; 95% CI -0.023−0.029; *P* = 0.83) (Figure 3B).

### Mortality

**Figure 4.**
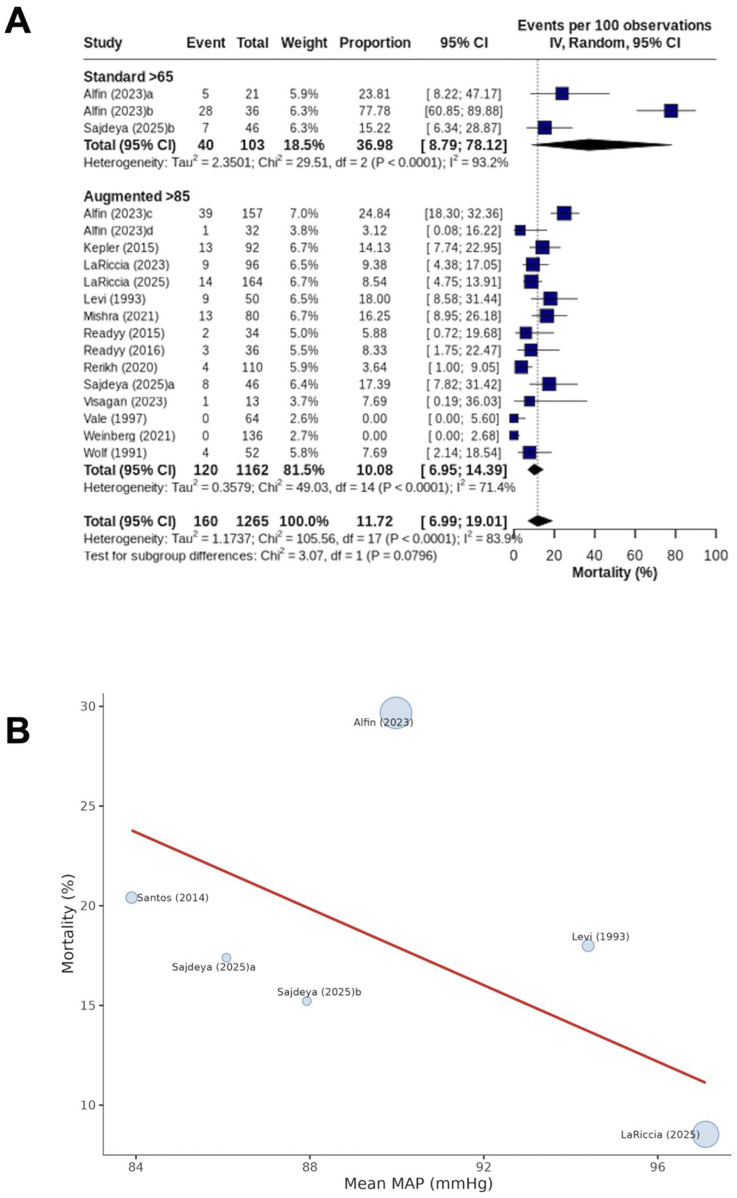
Mortality (A) Forest plot comparing (random effects model) mortality between patients subjected to standard MAP threshold and those undergoing MAP augmentation. Individual study estimates are shown as squares proportional to study weight. Horizontal lines indicate 95% confidence interval. Diamond represents pooled estimate for overall effect. IV = inverse variance; (B) Dose-response meta-regression for mortality, with MAP as the moderator

Fourteen studies^5,6,10,20,28,29,33-35,38-42^ (42.4%) reported mortality. Mortality occurred in 38.8% of patients subjected to the standard MAP threshold (40/103 patients) and 10.3% of patients subjected to MAP augmentation (120/1162 patients), with no significant difference between cohorts (*P* = 0.08; I^2^ = 83.9) (Figure 4A). Sensitivity analysis by treatment era did not suggest an effect of this moderator (Supplementary Figure 4). Similarly, cohort-level comparisons stratified by injury level and severity demonstrated no significant differences (Supplementary Figure 5). Dose-response meta-regression across 5 studies^5,10,20,28,43^ (15.2%) suggested that the MAP was not significantly associated with mortality (β = −0.96; 95% CI -2.95−1.03; *P* = 0.250) (Figure 4B).

### Complications

Six studies^6,26,33,34,39,40^ (18.2%) reported overall complications. Complications occurred in 54.9% of patients subjected to MAP augmentation (638/1163 patients) (Supplementary Figure 6). No study reported overall complications in patients subjected to the standard MAP threshold.

Respiratory complications were reported in 7 studies^10,32-35,38,39^ (21.2%) and occurred in 39.1% of patients subjected to the standard MAP threshold (18/46 patients) and 58.6% of patients subjected to MAP augmentation (191/326 patients). The proportion of respiratory complications was significantly lower in patients subjected to the standard MAP threshold (*P* = 0.04; I^2^ = 82.2) (Supplementary Figure 7). Cardiovascular complications were reported in 5 studies^26,28,33,34,44^ (15.2%) and occurred in 44.4% of patients subjected to MAP augmentation (514/1157 patients) (Supplementary Figure 8). Renal complications were reported in 6 studies^26,32-35,38^ (18.2%) and occurred in 20.5% of patients subjected to MAP augmentation (214/1046 patients) (Supplementary Figure 9). No studies reported cardiovascular or renal complications for patients subjected to standard MAP threshold.

### Length of Stay

Fifteen studies^10,20,21,25,28,29,33,34,39-42,45-47^ (45.5%) reported LOS. The mean LOS was 36.4 days in patients subjected to the standard MAP threshold and 22.5 days in patients subjected to MAP augmentation, with no significant difference between cohorts (*P* = 0.14; I^2^ = 96.0) (Supplementary Figure 10). Twelve studies^6,10,22,27-29,32-34,39,40,47^ (36.4%) reported ICU LOS. The mean LOS was 11.7 days in patients subjected to the standard MAP threshold and 8.3 days in patients subjected to MAP augmentation, with no significant difference between cohorts (*P* = 0.41; I^2^ = 99.3) (Supplementary Figure 11).

### Publication Bias, Risk of Bias, and Quality Assessment

Funnel plot inspection revealed no marked asymmetry, (Supplementary Figure 12). Egger’s regression test demonstrated no evidence of small-study effects (*P* = 0.245). Among the 33 included studies, most were judged to have a moderate risk, primarily due to residual confounding (Supplementary Table 4). Certainty of evidence for primary outcomes was judged to be low (Supplementary Table 5). Quality appraisal of 2 previous systematic reviews relevant to this topic were judged as critically low quality according to AMSTAR-2 criteria (Supplementary Table 6).

## Discussion

This systematic review and meta-analysis synthesizes data from 33 cohorts evaluating MAP management following acute traumatic SCI. In between-study, neurological improvement occurred in 31.4% of patients subjected to standard MAP and 37.6% in cohorts subjected to augmented (*P* = 0.25; I^2^ = 84.6). Mortality occurred in 38.8% and 10.3%, respectively (*P* = 0.08; I^2^ = 83.9), and achieved mean MAP differed modestly between cohorts (81.7 vs 90.0 mmHg). Meta-regression did not identify a linear association between achieved MAP and either primary outcome. Certainty of evidence for primary outcomes was low, limited primarily by inter-study heterogeneity, confounding by indication within studies, and ecological confounding (treatment era, case-mix, inclusion criteria) across studies. These findings should be viewed as associative rather than causal, and do not demonstrate the absence or presence of benefit from MAP augmentation in individual patients.

To date, the only level 1 evidence addressing this question is the RCT reported by Sajdeya and colleagues,^10^ which compared augmented (>85-90 mmHg) with conventional (>65-70 mmHg) MAP targets in 92 patients with acute traumatic SCI across 13 trauma centers. The trial found no significant difference in motor or sensory AIS recovery at 6 months, but did demonstrate more respiratory complications, longer mechanical ventilatory support, and higher organ dysfunction scores in the augmented arm. The observational evidence summarized in the present review should be interpreted in light of, and is broadly concordant with, this level 1 source. Across heterogeneous cohorts grouped by MAP target, we similarly observed no significant difference in neurological recovery, together with a significant excess of respiratory complication sin patients managed with augmented MAP. The counterintuitive crude mortality pattern (38.8% vs 10.3%) is unlikely to reflect a true treatment effect, given the asymmetric denominators (103 vs 1162), substantial interstudy heterogeneity (I^2^ = 83.9), and the absence of any moderator effect in era-stratified sensitivity analysis. These all argue against a causal interpretation, and the finding is most plausibly explained by study-level ecological confounding (treatment era, case-mix, inclusion criteria, and baseline severity).

As such, the principal contribution of this review is not to define a new MAP threshold, but to clarify why the existing literature remains difficult to interpret clinically: most studies were observational, achieved MAP separation between target cohorts was modest, vasopressor strategies remain heterogeneous, and neurological assessment timing ranged from 5 to 365 days.

### Contextualizing MAP Augmentation Within Its Evidentiary Origins

Traumatic SCI is commonly conceptualized as a biphasic process, comprising an immediate primary mechanical insult followed by a secondary injury cascade characterized by vascular dysfunction, ischemia, edema, and progressive cellular injury.^1,48^ Experimental pathology and physiological studies have consistently identified vascular compromise and reduced SCBF as central contributors to secondary injury progression^48-50^ even in the absence of overt mechanical vessel disruption.^51,52^ A key observation from this work is the loss of physiological autoregulation following trauma, rendering SCBF increasingly dependent on systemic perfusion pressure and providing the biological rationale for strict avoidance of hypotension in the acute phase.^3^

Initial clinical support for MAP augmentation was derived from small observational series employing invasive monitoring and protocolized cardiovascular support.^5,6,42,53-55^ These studies reported neurological outcomes that were considered encouraging in the context of severe injury, and subsequently informed guideline recommendations proposing maintenance of MAP at 85-90 mmHg for up to 7 days post-injury.^56,57^ Importantly, neither the numerical threshold nor duration was derived from controlled dose-response data, and these recommendations were explicitly framed as management options intended to buffer against impaired autoregulation, evolving cord edema and hemorrhage, and the risk of neurogenic shock, particularly in cervical and high-thoracic injuries.^58^ Despite expansion of the literature over subsequent decades, the empirical basis for these thresholds remains limited, and contemporary consensus statements continue to acknowledge uncertainty regarding optimal hemodynamic targets.^4,25,34^

### Interpreting the Absence of a Consistent Association With Outcome

In this context, the absence of a linear association between achieved MAP and neurological recovery or mortality observed in our dose-response meta-regression suggests that the escalation of systemic arterial pressure alone does not reliably translate into proportional improvements in effective spinal cord perfusion across heterogeneous injury states. A central assumption underlying MAP augmentation is that increasing systemic arterial pressure reliably improves perfusion at the site of maximal vulnerability within the injured spinal cord. Experimental data suggests that while MAP elevation may increase global SCBF, perfusion responses are spatially heterogeneous and may not uniformly benefit the injury epicenter, particularly after severe trauma. In such settings, blood flow may preferentially increase in less-injured regions, a phenomenon described as spatial perfusion “uncoupling”.^59^ Human physiological studies similarly suggest that increases in MAP do not necessarily translate into proportional increases in regional SCBF.^60,61^ When perfusion to the ischemic penumbra remains limited despite systemic hypertension, MAP augmentation is unlikely to meaningfully influence neurological recovery.^62^ While these mechanisms remain incompletely characterized in clinical populations, they offer a plausible explanation for the absence of consistent associations between higher MAP targets or achieved MAP and improved outcomes across observational cohorts.

### MAP as an Incomplete Surrogate for Spinal Cord Perfusion

An important limitation of MAP-centered strategies is that MAP represents only 1 determinant of SCPP. Following traumatic SCI, cord edema, hemorrhage, and swelling occur within a fixed dural compartment, leading to elevations in ISP.^63-65^ Under these conditions, SCPP, defined as MAP minus ISP, may more directly reflect effective tissue perfusion.^66^ Emerging observational studies suggest that SCPP may be more closely associated with neurological outcomes than MAP alone.^67-69^ However, ISP varies substantially between patients and dynamically over time, particularly in the early post-injury period.^24,25,29,64^ Consequently, a uniform MAP target may correspond to widely divergent SCPP values across individuals. Within this framework, it is plausible that some patients remain relatively hypoperfused despite guideline-concordant MAPs, whereas others are exposed to higher vasopressor doses without proportional perfusion benefit. These considerations highlight why fixed MAP thresholds may be physiologically imprecise, though definitive conclusions cannot be drawn from the available evidence.

### Timing, Variability, and Hypotensive Burden

Another challenge in interpreting MAP-based studies is the reliance on averaged MAP values over extended periods. Increasing evidence suggests that early perfusion status and burden of hypotensive episodes, particularly within the first 48-72 hours following injury, may be more relevant to secondary injury progression than averaged MAP values over several days.^24,25,29^ High-frequency physiological analyses indicate that transient hypotension may carry disproportionate prognostic value, with this effect appearing most pronounced in the early post-injury window.^21,25,39^ This distinction mirrors observations in other domains of vascular critical care, including stroke and traumatic brain injury, where avoidance of hypotension is consistently emphasized, but aggressive hypertensive strategies have not reliably demonstrated benefit. In SCI, the existing literature therefore supports the importance of hypotension avoidance but does not clearly establish that routine escalation beyond this threshold improves outcomes.

### Vasopressor Choice and Microvascular Considerations

MAP augmentation is achieved through diverse pharmacological strategies, and its physiological effects depend on the vasopressor employed. Experimental data suggest that agents differ in their effects on microvascular resistance and SCBF, with mixed α- and β-adrenergic agonists potentially exerting different perfusion effects compared with pure α-agonists.^70,71^ However, clinical studies evaluating vasopressor choice in SCI are sparse, heterogeneous, and confounded by injury severity and institutional practice.^72^ As such, while vasopressor selection may plausibly influence perfusion biology, the available evidence is insufficient to support agent-specific recommendations or to meaningfully stratify outcomes based on pharmacological strategy.

### Complications and Trade-offs of Aggressive Hemodynamic Support

The single statistically significant signal across all outcomes assessed in this review was an excess of respiratory complications in patients managed with augmented MAP (58.6% vs 39.1%; *P* = 0.04; I^2^ = 82.2%), a finding directly concordant with Sajdeya et al (2025),^10^ in which respiratory complications occurred in 78% of the augmented arm vs 39% of the conventional arm. Reporting of other complication categories across included studies was inconsistent, limiting quantitative synthesis of overall, cardiovascular, and renal complications. This association is biologically plausible given the vasopressor exposure and fluid administration often required to maintain elevated MAP targets, especially in patients with neurogenic shock. In the absence of consistent evidence for neurological benefit, these potential trade-offs warrant consideration, though causality cannot be inferred from the current data.

### Limitations of the Evidence Base

The conclusions of this review are constrained by quality and design of the underlying studies. Most included cohorts were observational and therefore subject to confounding by indication, which is particularly relevant in SCI complicated by neurogenic shock. Patients with cervical or high-thoracic injuries, who have a worse baseline prognosis, often require more aggressive vasopressor support to achieve prescribed MAP targets, such that observational comparisons may spuriously associate MAP augmentation with poorer outcomes. However, this mechanism does not explain the crude mortality pattern observed in this review, which is more consistent with study-level ecological confounding, including differences in treatment era, case-mix, inclusion criteria, and baseline injury severity. These factors preclude causal interpretation of the apparent mortality difference between cohorts.

Substantial heterogeneity was observed across primary outcomes. To explore sources of inter-study heterogeneity, prespecified exploratory analyses were performed stratifying cohorts by injury level and severity. However, heterogeneity largely persisted following stratification, suggesting that variability is not primarily driven by anatomical injury characteristics alone, but likely reflects broader inter-study differences, including MAP thresholds and achieved MAP values, monitoring fidelity, vasopressor choice, timing of neurological outcome assessment, and co-interventions across studies. Vasopressor strategies were heterogeneous and frequently multi-agent, precluding robust class- or agent-specific comparisons. Prescribed MAP thresholds frequently differed from achieved MAP values, as maintaining MAP within narrow target ranges is often impractical in the context of dynamic physiological fluctuations, complicating interpretation.^73^ In turn, the modest separation in achieved MAP between target cohorts (81.7 vs 90.0 mmHg) may have compromised the ability of study-level analyses to detect a therapeutic effect of MAP augmentation. Lastly, most studies reported AIS change as the primary neurological outcome, a relatively coarse measure that may not fully capture differences in recovery trajectory compared with more granular metrics (such as ASIA motor score). In addition, while a sensitivity analysis restricted to outcomes assessed at ≥ 3 months was feasible for the AIS improvement (proportion) outcome, the smaller number of studies and fragmentation of follow-up windows precluded an equivalent analysis for mean AIS improvement. This should be considered when interpreting the latter result.

LOS was a prespecified secondary outcome. However, hospital and ICU stay are highly multifactorial, reflecting injury severity, complications, surgical timing, regional rehabilitation pathways, and discharge logistics, and any observed differences across cohorts cannot be attributed to MAP management. The LOS findings reported here should therefore be regarded as descriptive only and do not inform conclusions regarding the clinical utility of MAP augmentation.

Given significant heterogeneity in baseline patient characteristics, exploratory meta-regression analyses using measured MAP were performed (adjusted for age, sex, injury level, and injury severity) for AIS improvement proportion (10 studies), mean AIS improvement (7 studies), and mortality (5 studies). Each of these analyses were informed by a limited number of contributing studies, and none was adequately powered to detect or exclude a clinically meaningful linear dose-response relationship between achieved MAP and the outcome assessed. Estimates should therefore be interpreted cautiously and viewed as exploratory rather than confirmatory. The absence of significant associations should not be construed as evidence of no underlying relationship. Finally, SCI is biologically heterogeneous, and neurological outcome may be strongly influenced by lesion location, tract involvement, and intrinsic plasticity, independent of perfusion physiology in some patients.^74^

## Conclusions

In summary, among cohorts grouped by MAP targets, we did not observe a significant pooled difference in neurological improvement or mortality. Interpretation remains limited by substantial heterogeneity, predominantly observational data, confounding by indication, and modest separation in achieved MAP. Current evidence supports strict avoidance of hypotension but does not establish that all patients benefit from routine augmentation to MAP. Future studies may benefit from physiology-informed approaches incorporating direct measures of ISP and SCPP, higher-resolution perfusion monitoring, and injury-specific stratification, but such strategies remain investigational at present.

## Supplemental Material

Supplemental Material - Mean Arterial Pressure Augmentation for Acute Traumatic Spinal Cord Injury: A Systematic Review and Meta-Analysis of Neurological Recovery and Mortality Supplemental Material for Mean Arterial Pressure Augmentation for Acute Traumatic Spinal Cord Injury: A Systematic Review and Meta-Analysis of Neurological Recovery and Mortality by Kush M. Kale, Shaan Patel, Shiva A. Nischal, Lorenzo Ceccon, Joshua Heller, Jack Jallo, James S. Harrop and Srinivas K. Prasad in Global Spine Journal

## Data Availability

No datasets were generated or analyzed during the current study.*

## Appendix

### Abbreviations

A MeaSurement Tool to Assess systematic Reviews 2 (AMSTAR-2)
American Spinal Injury Association (ASIA)
ASIA Impairment Scale (AIS)
Confidence Interval (CI)
Grading of Recommendations Assessment, Development and Evaluation (GRADE)
Intensive Care Unit (ICU)
Intraspinal Pressure (ISP)
Length of Stay (LOS)
Mean Arterial Pressure (MAP)
Mean Difference (MD)
Preferred Reporting Items for Systematic Reviews and Meta-Analyses (PRISMA)
Randomized Controlled Trial (RCT)
Restricted Maximum Likelihood (REML)
Risk of Bias 2 (RoB 2)
Risk of Bias in Non-randomised Studies of - Interventions Tool (ROBINS-I)
Risk Ratio (RR).
Spinal Cord Blood Flow (SCBF)
Spinal Cord Injury (SCI)
Spinal Cord Perfusion Pressure (SCPP).
