# Supplementary material for: Mean Arterial Pressure Augmentation for Acute Traumatic Spinal Cord Injury: A Systematic Review and Meta-Analysis of Neurological Recovery and Mortality
Source: Global Spine J. 2026 Jun 5:21925682261458879. Online ahead of print. doi: 10.1177/21925682261458879 (PMC13241385; doi:10.1177/21925682261458879)
Supplement: Supplemental Material - Mean Arterial Pressure Augmentation for Acute Traumatic Spinal Cord Injury: A Systematic Review and Meta-Analysis of Neurological Recovery and Mortality [file sj-pdf-1-gsj-10.1177_21925682261458879.pdf]

Study (year)	MAP target (mmHg)	Achieved MAP (mean ± SD) (mmHg)
Agarwal (2022)	76–104	NR
Alfin (2023)a	<80	90.0 ± 8.9
Alfin (2023)b	80–90	
Alfin (2023)c	90–100	
Alfin (2023)d	>100	
Balasuberamaniam (2023)	>80	NR
Catapano (2017)	>85	94.6 ± 2.8
Cohn (2010)	>65	NR
Dakson (2017)	>85	NR
Ehsanian (2020)	>70	78.3 ± 6.5
Haldrup (2020)	> 80	75.8 ± 12.6
Hawryluk (2015)	> 85	94.0 ± 1.2
Inoue (2014)	> 85	NR
Jiang (2019)	>85	NR
Kepler (2015)	> 85	NR
Langsjo (2024)a	65–85	81.6 ± 3.9
Langsjo (2024)b	85–90	90.2 ± 2.2
LaRiccia (2023)	>85	NR
LaRiccia (2025)	>85	97.1 ± 17.7
Levi (1993)	>90	94.4 ± 9.4
Martin (2015)	>85	85.0 ± 6.4
Mishra (2021)	>85	NR
Mushlin (2020)	>85	89.0 ± 5.7
Park (2016)	>85	NR
Rask (2024)	>85	81.0 ± 10.3
Readyy (2015)	>85	NR
Readyy (2016)	>85	NR
Rerikh (2020)	>85–90	NR
Sajdeya (2025)a	>85–90	86.1 ± 11.9
Sajdeya (2025)b	>65–70	87.9 ± 14.0
Santos (2014)	NR	83.9
Sewell (2019)	>80	NR
Squair (2019)	>80–85	85.0 ± 6.0
Visagan (2023)	>85	NR
Vale (1997)	>85	NR
Weinberg (2021)	>85	NR
Wolf (1991)	>85	NR
Zhang (2024)	>70	NR

Supplementary Table 2: Summary of target and achieved MAP

Target and achieved MAP for each included study in the present meta-analysis. MAP, mean arterial pressure; NR, not reported.

No.	Item	Outcome
1	Did the research questions and inclusion criteria for the review include the components of PICO?	Y
2	*Did the report of the review contain an explicit statement that the review methods were established prior to the conduct of the review and did the report justify any significant deviations from the protocol?	Y
3	Did the review authors explain their selection of the study designs for inclusion in the review?	Y
4	*Did the review authors use a comprehensive literature search strategy?	Y
5	Did the review authors perform study selection in duplicate?	Y
6	Did the review authors perform data extraction in duplicate?	Y
7	*Did the review authors provide a list of excluded studies and justify the exclusions?	Y
8	Did the review authors describe the included studies in adequate detail?	Y
9	*Did the review authors use a satisfactory technique for assessing the risk of bias (RoB) in individual studies that were included in the review?	Y
10	Did the review authors report on the sources of funding for the studies included in the review?	Y
11	*If meta-analysis was performed did the review authors use appropriate methods for statistical combination of results?	Y
12	If meta-analysis was performed, did the review authors assess the potential impact of RoB in individual studies on the results of the meta-analysis or other evidence synthesis?	Y
13	*Did the review authors account for RoB in individual studies when interpreting/ discussing the results of the review?	Y
14	Did the review authors provide a satisfactory explanation for, and discussion of, any heterogeneity observed in the results of the review?	Y
15	*If they performed quantitative synthesis did the review authors carry out an adequate investigation of publication bias (small study bias) and discuss its likely impact on the results of the review?	Y
16	Did the review authors report any potential sources of conflict of interest, including any funding they received for conducting the review?	Y

Supplementary Table 3: AMSTAR-2 quality appraisal
AMSTAR-2 criteria applied to the present review, with item-level judgements (Y = yes; PY = partial yes; N = no). *= Critical weaknesses

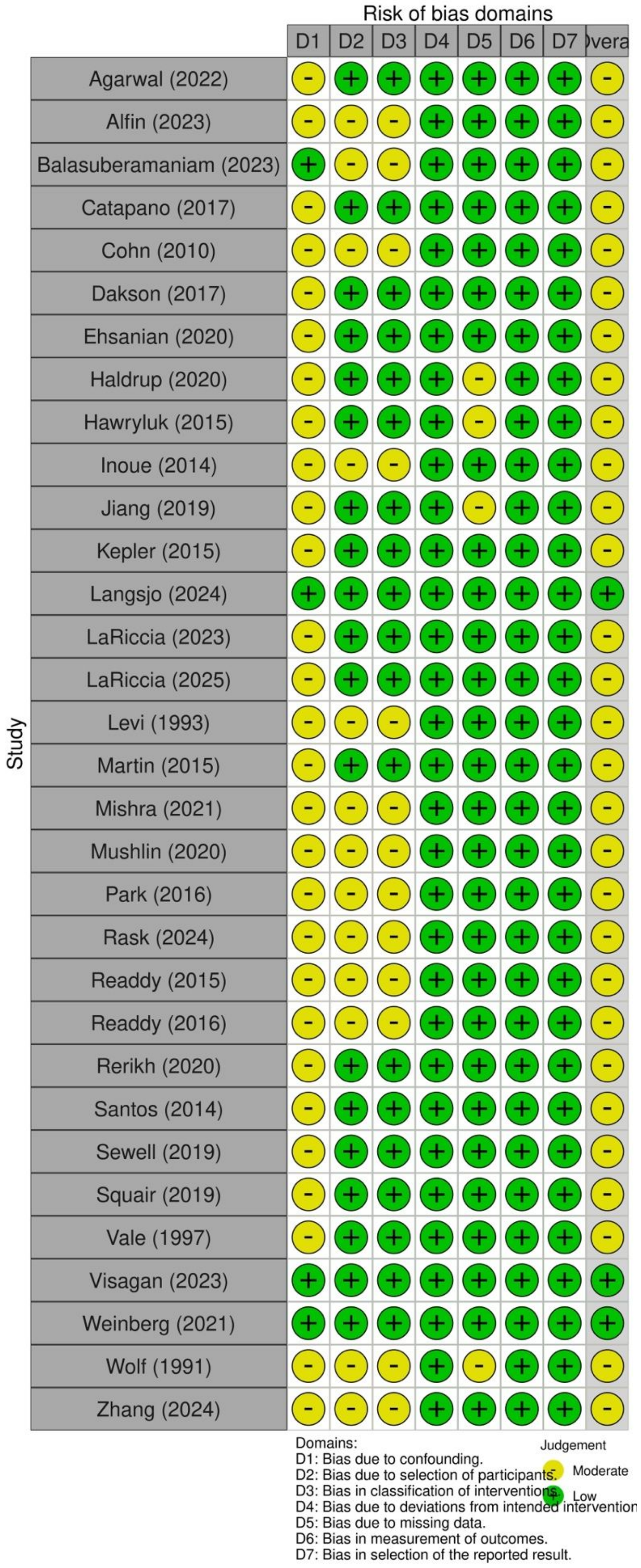

Supplementary Table 4A: Risk of bias assessment (non-randomised)

Summary of risk of bias assessment for non-randomised studies (ROBINS-I), reported by individual domain and overall risk of bias.

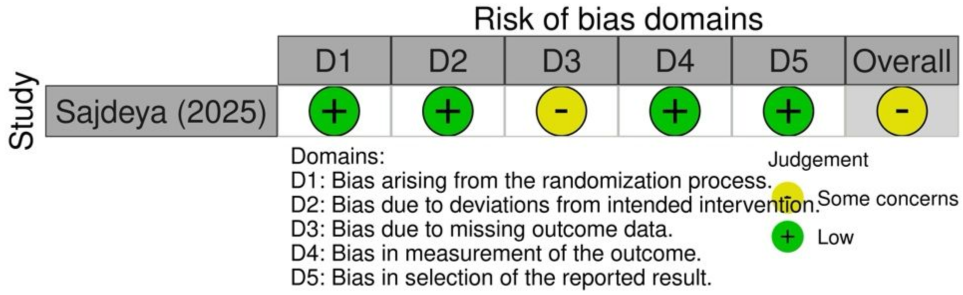

Supplementary Table 4B: Risk of bias assessment (randomised)
Summary of risk of bias assessment for randomised studies (RoB 2), reported by individual domain and overall risk of bias.

No. of studies	Risk of bias	Inconsistency, indirectness, imprecision	Other Considerations	No. patients	Summary of findings		Certainty (GRADE)
					Standard	Augmented	
**AIS Improvement (Proportion)**							
23	Serious	Serious (inconsistency)	None	2093	160/509	595/1584	Low
**AIS Improvement (Mean)**							
17	Serious	Serious (inconsistency)	None	1239	0.48 grades	0.55 grades	Low
**Mortality**							
14	Serious	Serious (inconsistency)	None	1265	40/103	120/1162	Low

Supplementary Table 5: Certainty of evidence (GRADE)
GRADE assessment of certainty of evidence for each primary outcome in the present review.

Author	Critical Flaws	Non-Critical Flaws	Overall Confidence
Saadeh et al. (2017)	**Item 2** - No explicit protocol registered before conducting the review<br>**Item 4** - Non-comprehensive search strategy (PubMed only)<br>**Item 7** - No list of excluded studies provided<br>**Item 9** - No risk of bias assessment performed<br>**Item 13** - Risk of bias not considered in results<br>**Item 15** - Publication bias not considered	**Item 5** - No explicit statement that study selection was performed in duplicate<br>**Item 6** - No explicit statement that data extraction was performed in duplicate<br>**Item 10** - Funding sources of included studies not reported<br>**Item 12** - Did not explore impact of RoB	**Critically Low**
Tanvir et al. (2025)	**Item 9** - Inappropriate risk of bias assessment performed (used Newcastle-Ottawa Scale)	**Item 10** - Funding sources of included studies not reported	**Critically Low**
Kale & Patel et al., 2026 (this review)	**None**	**None**	**High**

Supplementary Table 6: AMSTAR-2 quality appraisal of prior reviews
Summary of AMSTAR-2 assessment of previously published systematic reviews on this topic, indicating critical and non-critical flaws, and overall confidence rating.

Study	Improved	Total	Weight	Proportion	95% CI
**Standard (>65) - Post 2010**					
Agarwal (2022)	38	74	4.1%	51.35	[39.44; 63.15]
Alfin (2023)a	3	16	2.5%	18.75	[4.05; 45.65]
Alfin (2023)b	7	58	3.4%	12.07	[4.99; 23.30]
Cohn (2010)	2	17	2.1%	11.76	[1.46; 36.44]
Langsjo (2024)a	9	19	3.2%	47.37	[24.45; 71.14]
Haldrup (2020)	35	129	4.3%	27.13	[19.68; 35.66]
Sajdeya (2025)b	11	25	3.4%	44.00	[24.40; 65.07]
Sewell (2019)	12	79	3.8%	15.19	[8.10; 25.03]
Squair (2019)	43	92	4.2%	46.74	[36.26; 57.44]
**Total (95% CI)**	**160**	**509**	**31.1%**	**29.41**	**[19.30; 42.06]**
Heterogeneity: $Tau^2 = 0.5597$; $Chi^2 = 48.19$, df = 8 ($P < 0.0001$); $I^2 = 83.4\%$					
**Augmented (>85) - Post 2010**					
Alfin (2023)c	33	118	4.2%	27.97	[20.10; 36.98]
Alfin (2023)d	4	31	2.9%	12.90	[3.63; 29.83]
Catapano (2017)	31	62	4.1%	50.00	[37.02; 62.98]
Dakson (2017)	12	61	3.8%	19.67	[10.60; 31.84]
Hawryluk (2015)	36	71	4.1%	50.70	[38.56; 62.78]
Jiang (2019)	163	344	4.5%	47.38	[42.00; 52.81]
Langsjo (2024)b	19	32	3.6%	59.38	[40.64; 76.30]
LaRiccia (2023)	14	96	3.9%	14.58	[8.21; 23.26]
LaRiccia (2025)	36	164	4.3%	21.95	[15.87; 29.07]
Mushlin (2020)	39	73	4.1%	53.42	[41.37; 65.20]
Park (2016)	27	73	4.1%	36.99	[25.97; 49.09]
Rask (2024)	10	40	3.6%	25.00	[12.69; 41.20]
Readyy (2015)	19	34	3.7%	55.88	[37.89; 72.81]
Readyy (2016)	9	36	3.5%	25.00	[12.12; 42.20]
Rerikh (2020)	42	110	4.3%	38.18	[29.08; 47.93]
Sajdeya (2025)a	15	27	3.5%	55.56	[35.33; 74.52]
Visagan (2023)	9	12	2.4%	75.00	[42.81; 94.51]
Weinberg (2021)	40	136	4.3%	29.41	[21.91; 37.83]
**Total (95% CI)**	**558**	**1520**	**68.9%**	**36.82**	**[29.32; 45.02]**
Heterogeneity: $Tau^2 = 0.4451$; $Chi^2 = 113.81$, df = 17 ($P < 0.0001$); $I^2 = 85.1\%$					
**Total (95% CI)**	**718**	**2029**	**100.0%**	**34.50**	**[28.30; 41.28]**
Heterogeneity: $Tau^2 = 0.4696$; $Chi^2 = 164.53$, df = 26 ($P < 0.0001$); $I^2 = 84.2\%$					
Test for subgroup differences: $Chi^2 = 1.02$, df = 1 ($P = 0.3119$)					

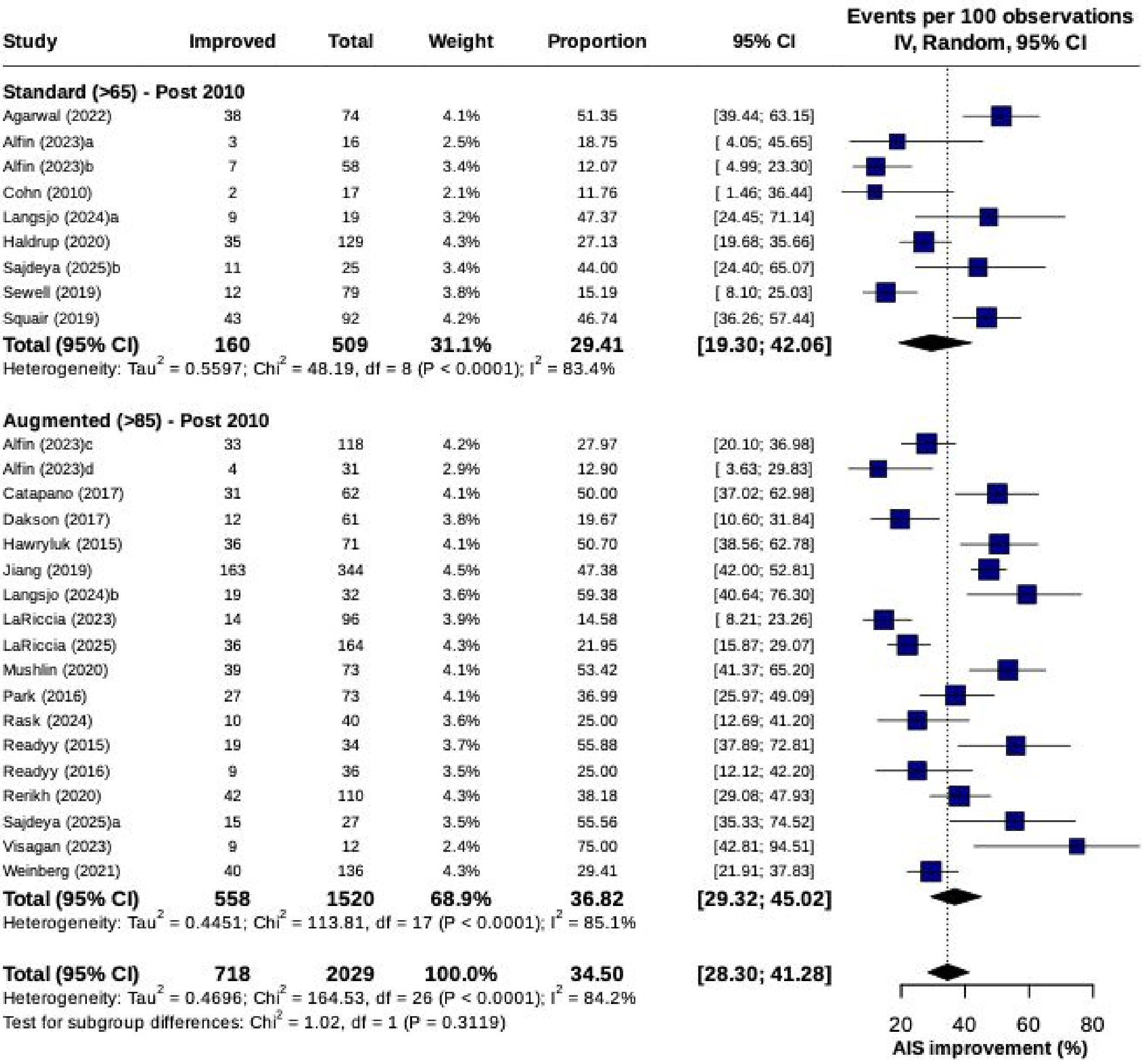

Supplementary Figure 1: AIS Improvement Proportion (Sensitivity Analysis by Treatment Era)
Forest plots reporting the proportion of patients experiencing AIS grade improvement, with cohort-level comparison (random effects model) by MAP target and treatment era[1]. Individual study estimates are shown as squares proportional to study weight. Horizontal lines indicate 95% confidence interval. Diamond represents pooled estimate for overall effect. GLMM = generalized linear mixed model.

[1]Cohorts were grouped by protocolized MAP target. Comparisons therefore represent between-cohort, single-arm analyses rather than within-study comparisons.

Study	Improved	Total	Weight	Proportion	95% CI
**Standard (>65) - <3 months**					
Agarwal (2022)	38	74	4.0%	51.35	[39.44; 63.15]
Alfin (2023)a	3	16	2.4%	18.75	[4.05; 45.65]
Alfin (2023)b	7	58	3.3%	12.07	[4.99; 23.30]
Cohn (2010)	2	17	2.0%	11.76	[1.46; 36.44]
Sewell (2019)	12	79	3.7%	15.19	[8.10; 25.03]
**Total (95% CI)**	**62**	**244**	**15.4%**	**20.64**	**[9.94; 37.98]**
Heterogeneity: $Tau^2 = 0.7333$; $Chi^2 = 33.89$, df = 4 ($P < 0.0001$); $I^2 = 88.2\%$					
**Augmented (>85) - <3 months**					
Alfin (2023)c	33	118	4.1%	27.97	[20.10; 36.98]
Alfin (2023)d	4	31	2.8%	12.90	[3.63; 29.83]
Catapano (2017)	31	62	3.9%	50.00	[37.02; 62.98]
Hawryluk (2015)	36	71	4.0%	50.70	[38.56; 62.78]
Langsjo (2024)b	19	32	3.5%	59.38	[40.64; 76.30]
LaRiccia (2023)	14	96	3.8%	14.58	[8.21; 23.26]
LaRiccia (2025)	36	164	4.1%	21.95	[15.87; 29.07]
Readyy (2015)	19	34	3.5%	55.88	[37.89; 72.81]
Readyy (2016)	9	36	3.4%	25.00	[12.12; 42.20]
Weinberg (2021)	40	136	4.1%	29.41	[21.91; 37.83]
**Total (95% CI)**	**241**	**780**	**37.1%**	**33.10**	**[23.24; 44.71]**
Heterogeneity: $Tau^2 = 0.5351$; $Chi^2 = 64.26$, df = 9 ($P < 0.0001$); $I^2 = 86\%$					
**Standard (>65) - ≥3 months**					
Langsjo (2024)a	9	19	3.1%	47.37	[24.45; 71.14]
Haldrup (2020)	35	129	4.1%	27.13	[19.68; 35.66]
Sajdeya (2025)b	11	25	3.3%	44.00	[24.40; 65.07]
Squair (2019)	43	92	4.1%	46.74	[36.26; 57.44]
**Total (95% CI)**	**98**	**265**	**14.5%**	**39.58**	**[28.68; 51.63]**
Heterogeneity: $Tau^2 = 0.1543$; $Chi^2 = 10.34$, df = 3 ($P = 0.0159$); $I^2 = 71\%$					
**Augmented (>85) - ≥3 months**					
Dakson (2017)	12	61	3.6%	19.67	[10.60; 31.84]
Jiang (2019)	163	344	4.3%	47.38	[42.00; 52.81]
Mushlin (2020)	39	73	4.0%	53.42	[41.37; 65.20]
Park (2016)	27	73	3.9%	36.99	[25.97; 49.09]
Rask (2024)	10	40	3.5%	25.00	[12.69; 41.20]
Rerikh (2020)	42	110	4.1%	38.18	[29.08; 47.93]
Sajdeya (2025)a	15	27	3.4%	55.56	[35.33; 74.52]
Visagan (2023)	9	12	2.3%	75.00	[42.81; 94.51]
Vale (1997)	37	64	3.9%	57.81	[44.82; 70.06]
**Total (95% CI)**	**354**	**804**	**33.0%**	**43.47**	**[33.48; 54.02]**
Heterogeneity: $Tau^2 = 0.3261$; $Chi^2 = 35.96$, df = 8 ($P < 0.0001$); $I^2 = 77.8\%$					
**Total (95% CI)**	**755**	**2093**	**100.0%**	**35.34**	**[29.09; 42.13]**
Heterogeneity: $Tau^2 = 0.4849$; $Chi^2 = 175.31$, df = 27 ($P < 0.0001$); $I^2 = 84.6\%$					
Test for subgroup differences: $Chi^2 = 5.68$, df = 3 ($P = 0.1281$)					

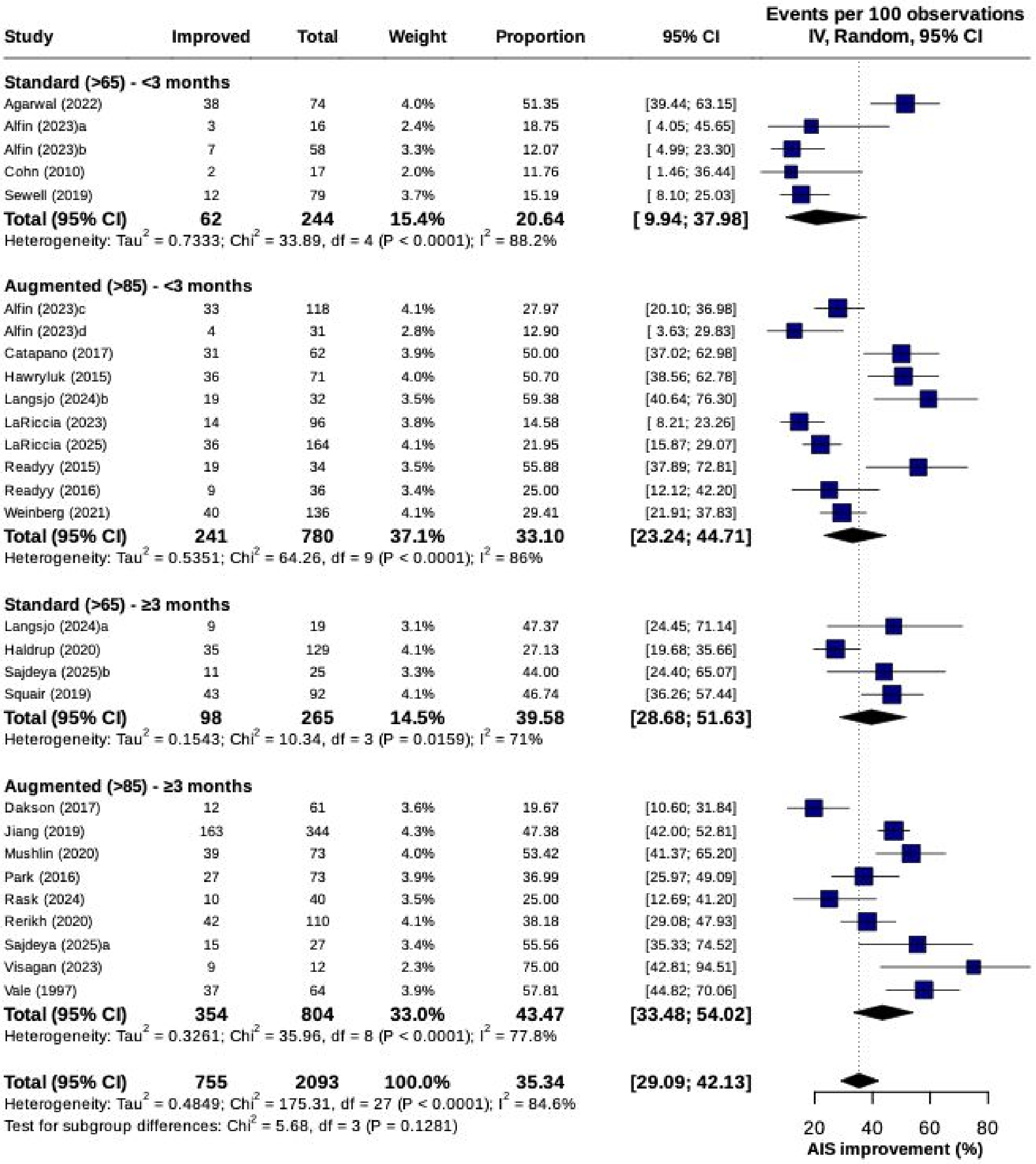

Supplementary Figure 2: AIS Improvement Proportion (Sensitivity Analysis by Follow-Up Duration) Forest plots reporting the proportion of patients experiencing AIS grade improvement, with cohort-level comparison (random effects model) by MAP target and follow-up duration[1]. Individual study estimates are shown as squares proportional to study weight. Horizontal lines indicate 95% confidence interval. Diamond represents pooled estimate for overall effect. GLMM = generalized linear mixed model.

[1]Cohorts were grouped by protocolized MAP target. Comparisons therefore represent between-cohort, single-arm analyses rather than within-study comparisons.

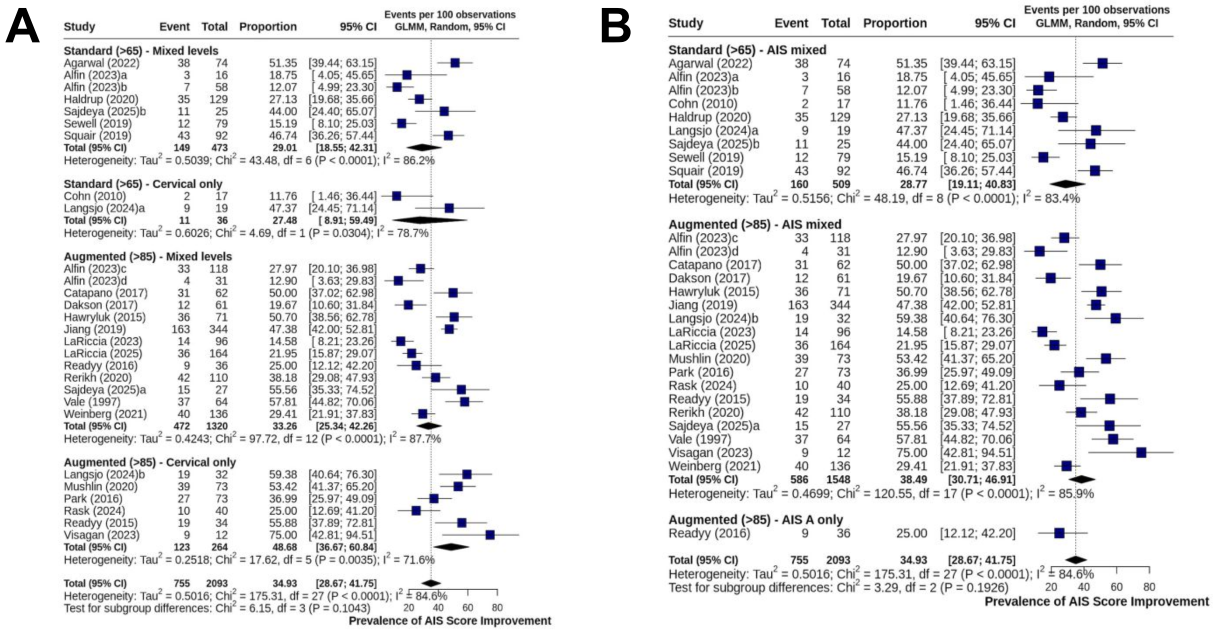

Supplementary Figure 3: AIS Improvement Proportion (Exploratory Analyses)
Forest plots reporting the proportion of patients experiencing AIS grade improvement, with cohort-level comparison (random effects model) by MAP target and (A) injury level or (B) injury severity[1]. Individual study estimates are shown as squares proportional to study weight. Horizontal lines indicate 95% confidence interval. Diamond represents pooled estimate for overall effect. GLMM = generalized linear mixed model.

[1]Cohorts were grouped by protocolized MAP target. Comparisons therefore represent between-cohort, single-arm analyses rather than within-study comparisons.

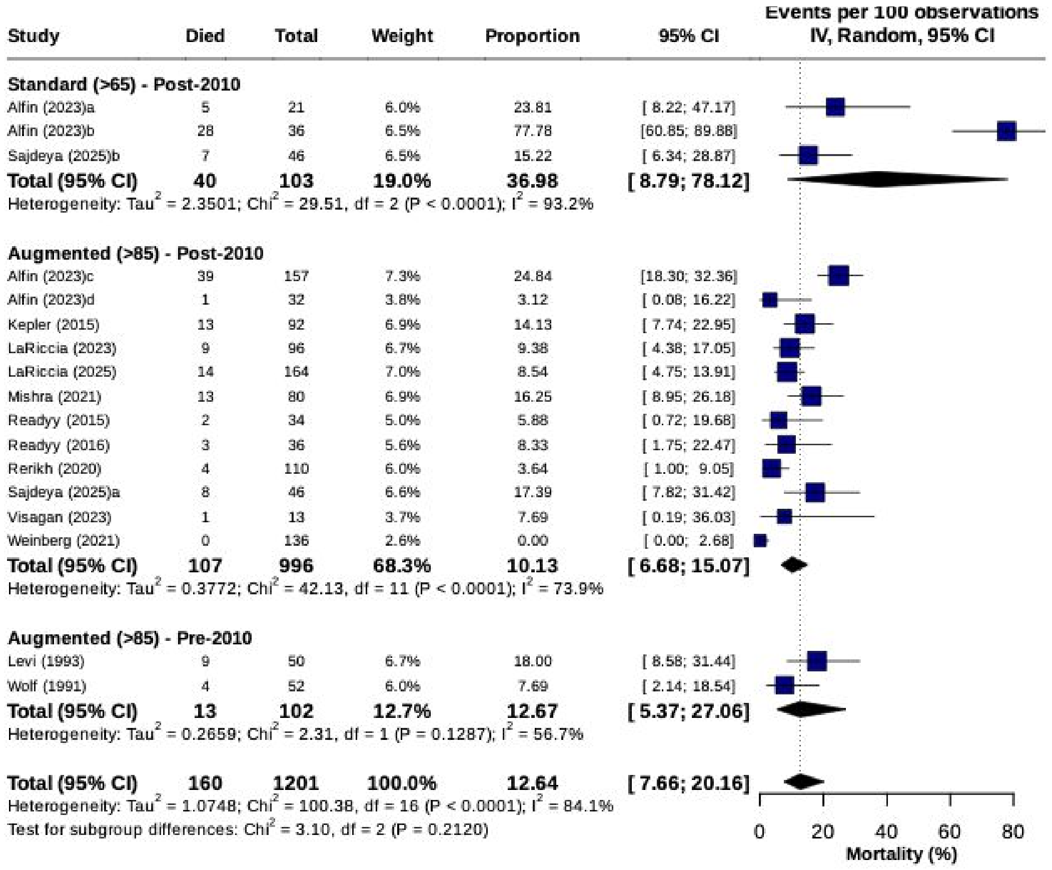

Supplementary Figure 4: Mortality (Sensitivity Analysis by Treatment Era)
Forest plots reporting mortality, with cohort-level comparison (random effects model) by MAP target and treatment era[1]. Individual study estimates are shown as squares proportional to study weight. Horizontal lines indicate 95% confidence interval. Diamond represents pooled estimate for overall effect. GLMM = generalized linear mixed model.

[1]Cohorts were grouped by protocolized MAP target. Comparisons therefore represent between-cohort, single-arm analyses rather than within-study comparisons.

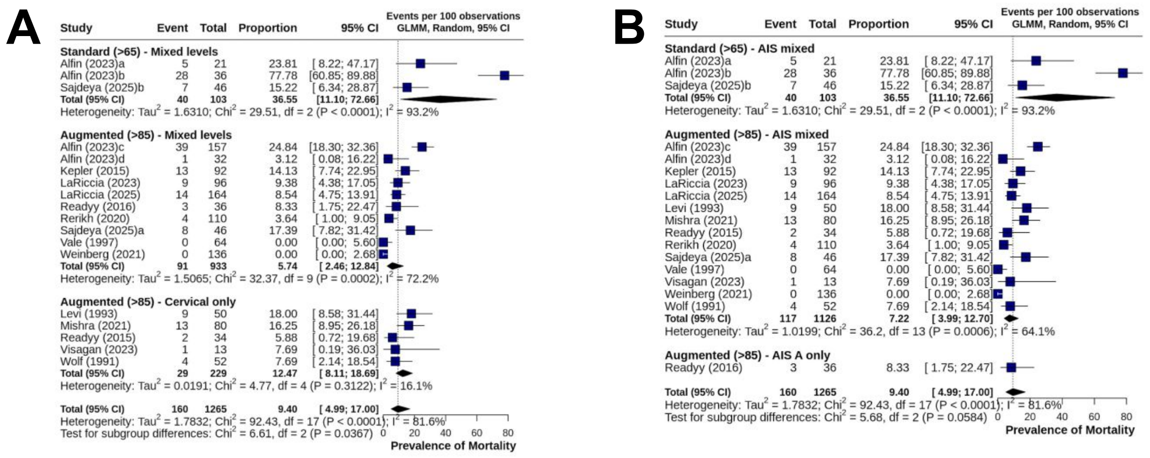

Supplementary Figure 5: Mortality (Exploratory Analyses)

Forest plots reporting the mortality, with cohort-level comparison (random effects model) by MAP target and (A) injury level or (B) injury severity[1]. Individual study estimates are shown as squares proportional to study weight. Horizontal lines indicate 95% confidence interval. Diamond represents pooled estimate for overall effect. GLMM = generalized linear mixed model.

[1]Cohorts were grouped by protocolized MAP target. Comparisons therefore represent between-cohort, single-arm analyses rather than within-study comparisons.

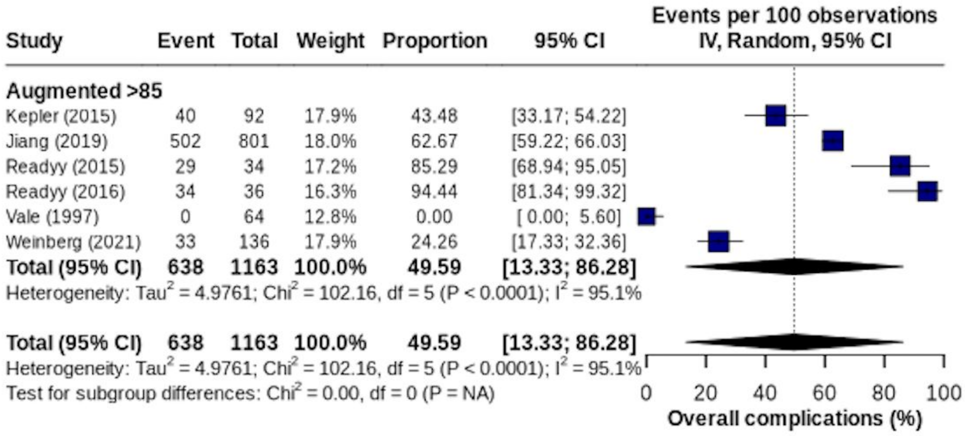

Supplementary Figure 6: Overall Complications

Forest plot reporting the incidence (random effects model) of overall complications in patients undergoing MAP augmentation. Individual study estimates are shown as squares proportional to study weight. Horizontal lines indicate 95% confidence interval. Diamond represents pooled estimate for overall effect. IV = inverse variance.

Study	Event	Total	Weight	Proportion	95% CI
**Standard >65**					
Sajdeya (2025)b	18	46	13.3%	39.13	[25.09; 54.63]
**Augmented >85**					
Rask (2024)	19	52	13.4%	36.54	[23.62; 51.04]
Readyy (2015)	27	34	11.7%	79.41	[62.10; 91.30]
Readyy (2016)	29	36	11.8%	80.56	[63.98; 91.81]
Rerikh (2020)	57	110	14.4%	51.82	[42.09; 61.45]
Sajdeya (2025)a	36	45	12.4%	80.00	[65.40; 90.42]
Visagan (2023)	7	13	10.1%	53.85	[25.13; 80.78]
Weinberg (2021)	16	36	12.9%	44.44	[27.94; 61.90]
**Total (95% CI)**	**191**	**326**	**86.7%**	**61.91**	**[46.30; 75.40]**
Heterogeneity: $Tau^2 = 0.5929$; $Chi^2 = 34.35$, df = 6 ($P < 0.0001$); $I^2 = 82.5\%$					
**Total (95% CI)**	**209**	**372**	**100.0%**	**58.97**	**[44.31; 72.19]**
Heterogeneity: $Tau^2 = 0.5947$; $Chi^2 = 39.24$, df = 7 ($P < 0.0001$); $I^2 = 82.2\%$					
Test for subgroup differences: $Chi^2 = 4.39$, df = 1 ($P = 0.0361$)					

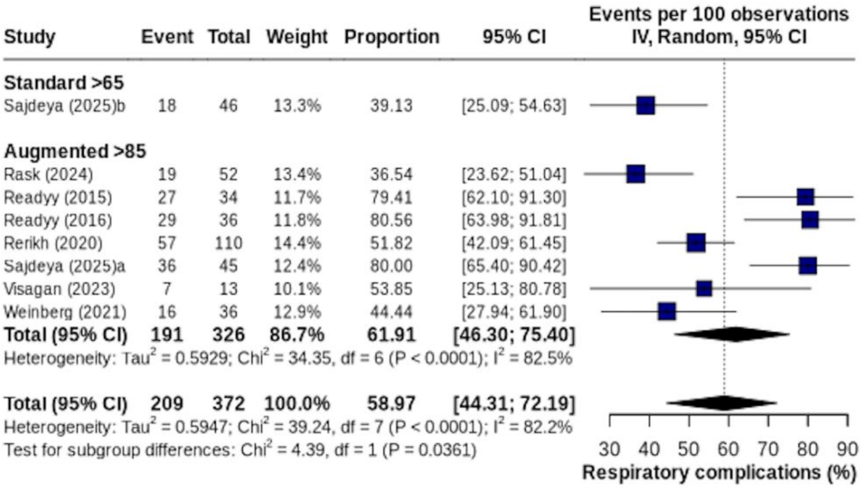

Supplementary Figure 7: Respiratory Complications

Forest plot comparing the incidence of respiratory complications, with cohort-level comparison (random effects model) by MAP target and (A) injury level or (B) injury severity[1]. Individual study estimates are shown as squares proportional to study weight. Horizontal lines indicate 95% confidence interval. Diamond represents pooled estimate for overall effect. IV = inverse variance.

[1]Cohorts were grouped by protocolized MAP target. Comparisons therefore represent between-cohort, single-arm analyses rather than within-study comparisons.

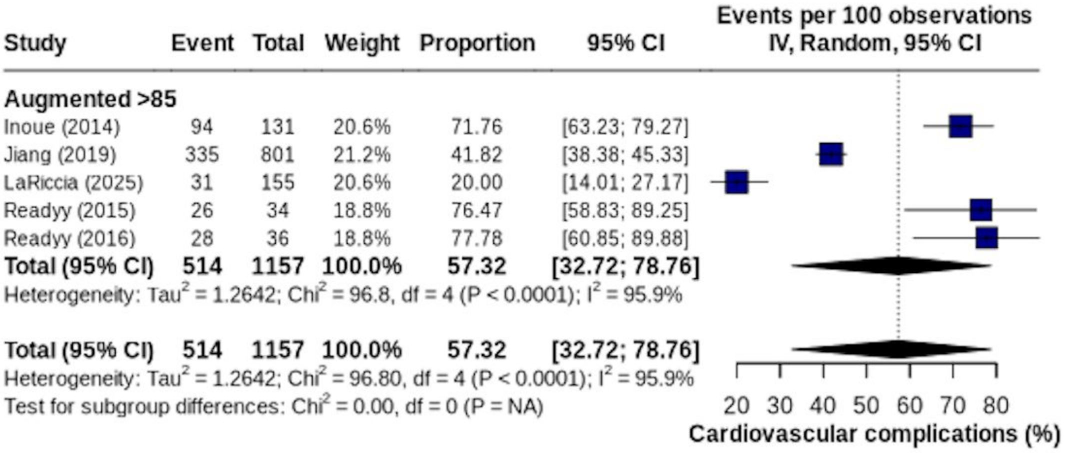

Supplementary Figure 8: Cardiovascular Complications

Forest plot reporting the incidence (random effects model) of cardiovascular complications in patients undergoing MAP augmentation. Individual study estimates are shown as squares proportional to study weight. Horizontal lines indicate 95% confidence interval. Diamond represents pooled estimate for overall effect. IV = inverse variance.

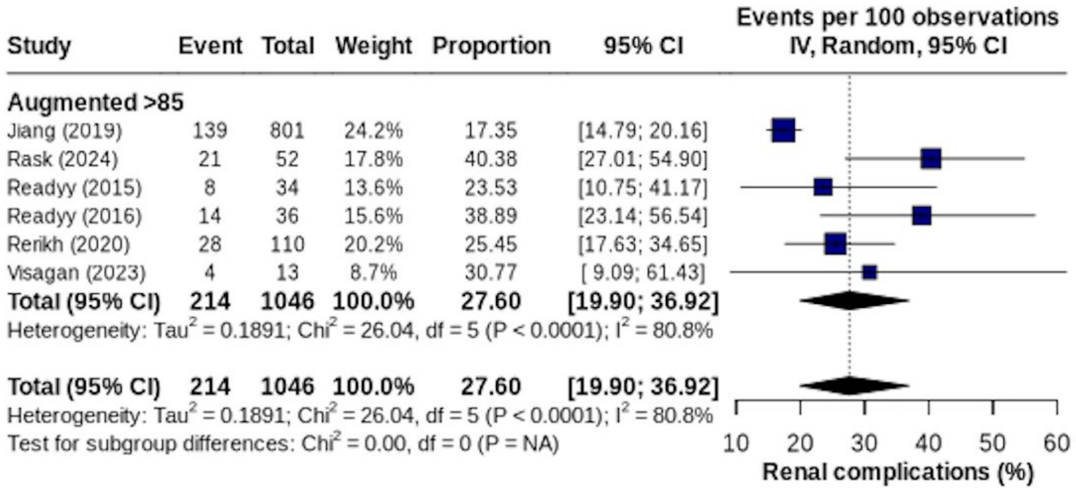

Supplementary Figure 9: Renal Complications

Forest plot reporting the incidence (random effects model) of renal complications in patients undergoing MAP augmentation. Individual study estimates are shown as squares proportional to study weight. Horizontal lines indicate 95% confidence interval. Diamond represents pooled estimate for overall effect. IV = inverse variance.

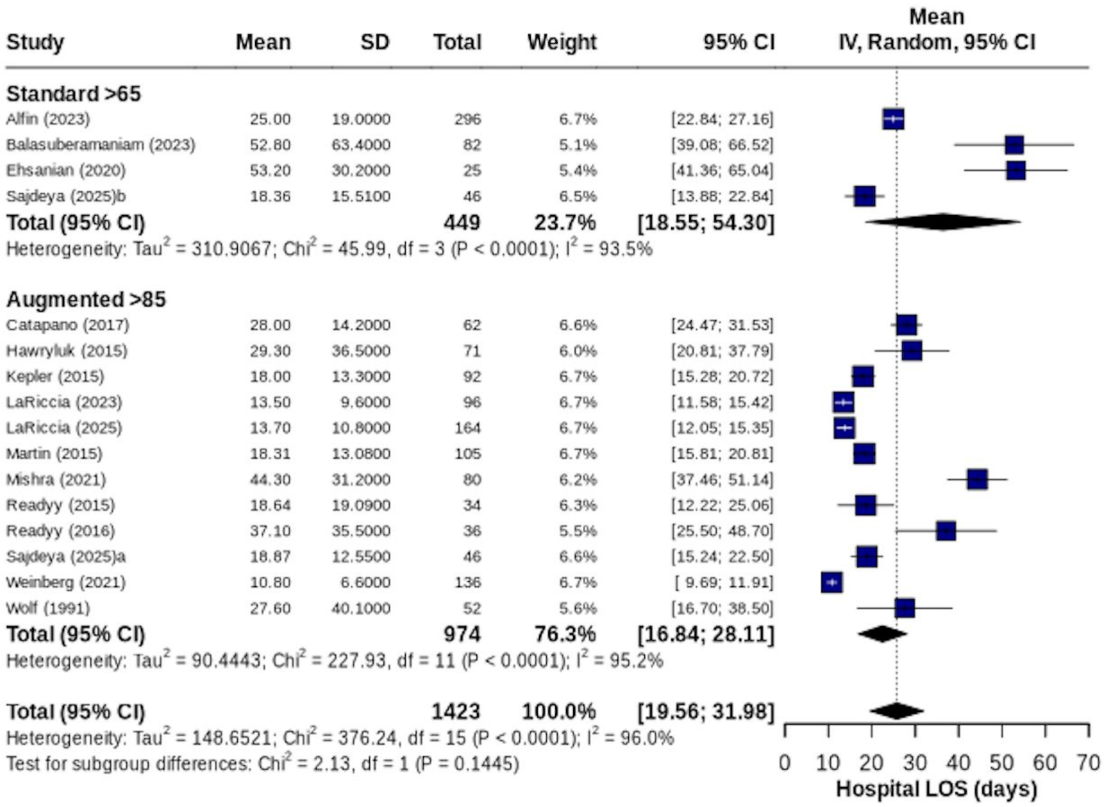

Supplementary Figure 10: Hospital length of stay

Forest plot reporting mean hospital length of stay (LOS), with cohort-level comparison (random effects model) by MAP target and (A) injury level or (B) injury severity[1]. Individual study estimates are shown as squares proportional to study weight. Horizontal lines indicate 95% confidence interval. Diamond represents pooled estimate for overall effect. IV = inverse variance.

[1]Cohorts were grouped by protocolized MAP target. Comparisons therefore represent between-cohort, single-arm analyses rather than within-study comparisons.

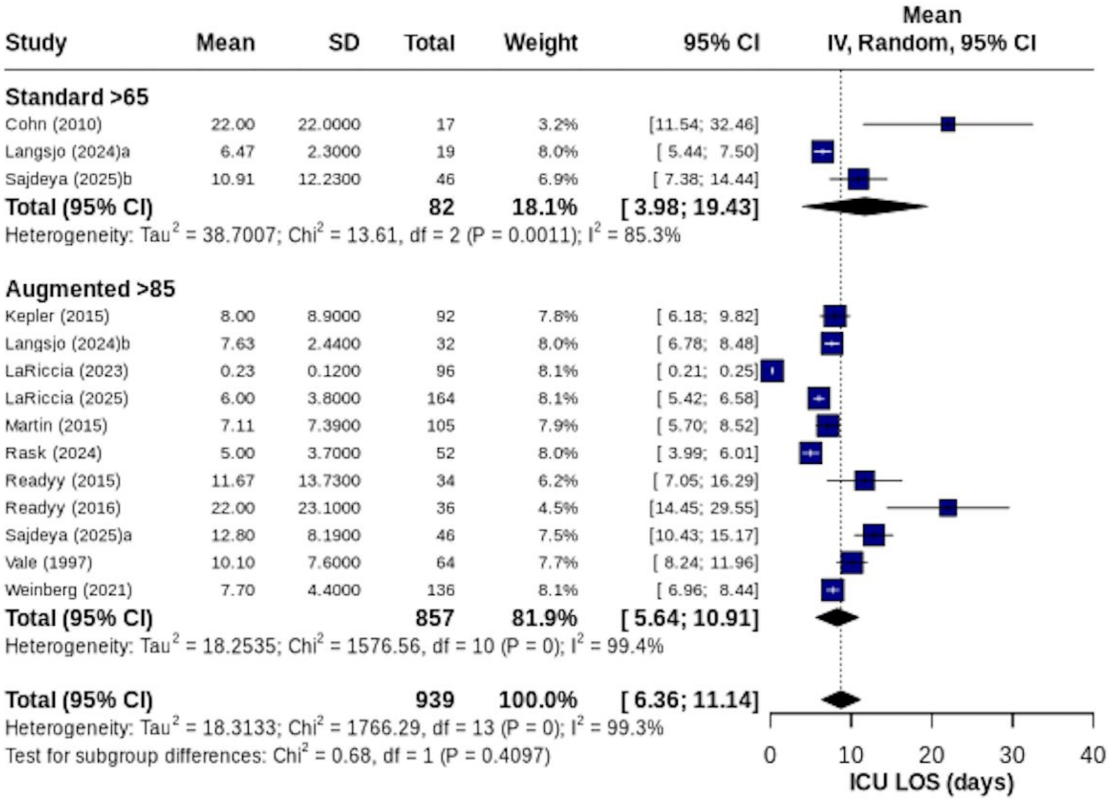

Supplementary Figure 11: ICU length of stay

Forest plot reporting mean ICU length of stay, with cohort-level comparison (random effects model) by MAP target and (A) injury level or (B) injury severity[1]. Individual study estimates are shown as squares proportional to study weight. Horizontal lines indicate 95% confidence interval. Diamond represents pooled estimate for overall effect. IV = inverse variance.

[1]Cohorts were grouped by protocolized MAP target. Comparisons therefore represent between-cohort, single-arm analyses rather than within-study comparisons.

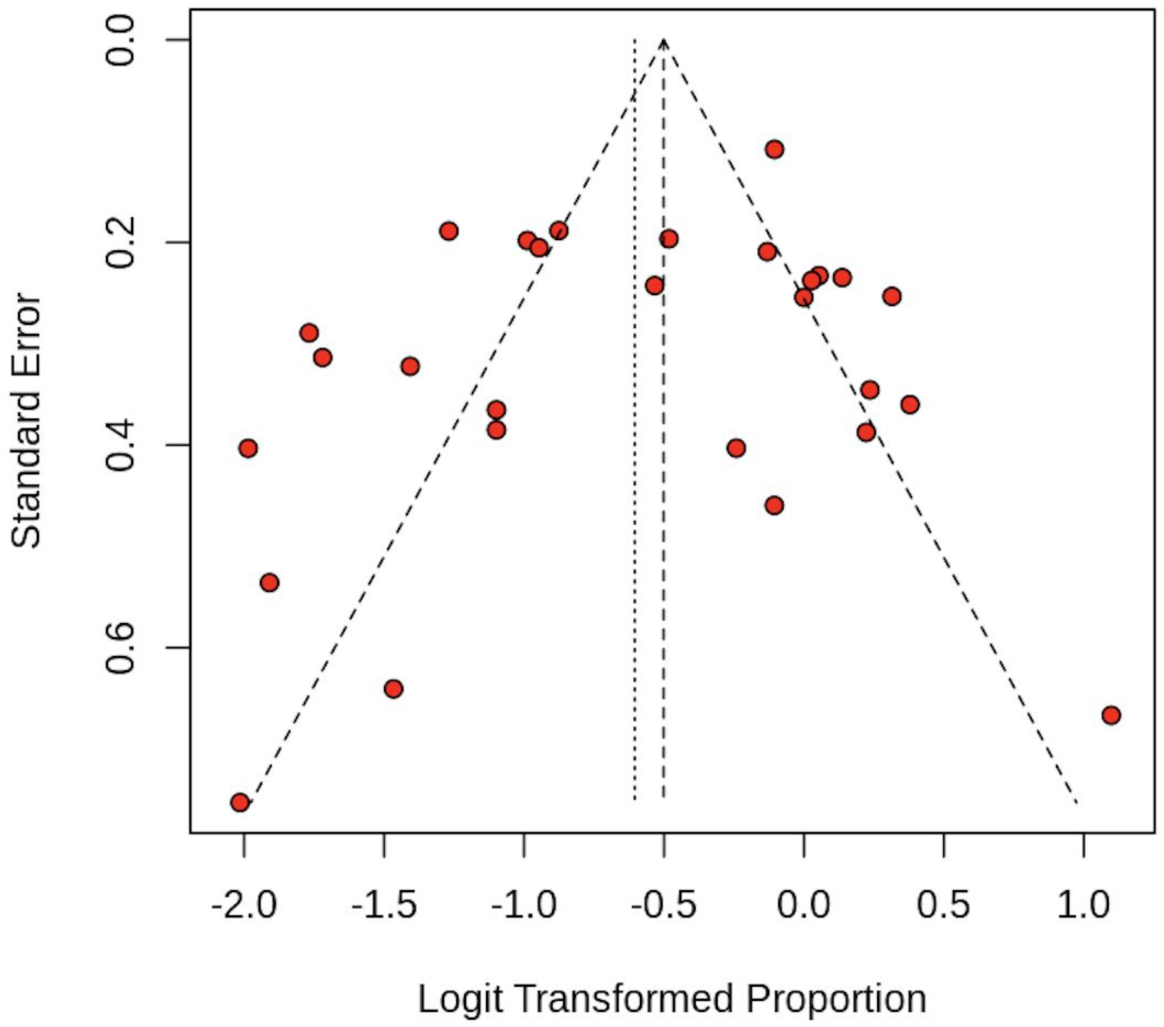

Supplementary Figure 12: Publication bias assessment

Funnel plot of effect estimates from included studies plotted against their respective standard errors for the outcome of AIS improvement (proportion).
